# Regulation of leukotriene and 5oxoETE synthesis and the effect of 5-lipoxygenase inhibitors: a mathematical modeling approach

**DOI:** 10.1186/1752-0509-6-141

**Published:** 2012-11-12

**Authors:** Tatiana A Karelina, Kirill V Zhudenkov, Oleg O Demin, Dmitry V Svetlichny, Balaji Agoram, David Fairman, Oleg V Demin

**Affiliations:** 1 Institute for Systems biology SPb, Moscow, Russia; 2 Clinical Pharmacology, Pfizer PGRD, Sandwich, UK; 3 Now Clinical Pharmacology and DMPK, MedImmune, Cambridge, UK; 4 Pharmacokinetics, Dynamics and Metabolism, Pfizer PGRD, Sandwich, UK

## Abstract

**Background:**

5-lipoxygenase (5-LO) is a key enzyme in the synthesis of leukotrienes and 5-Oxo-6E,8Z,11Z,14Z-eicosatetraenoic acid (oxoETE). These inflammatory signaling molecules play a role in the pathology of asthma and so 5-LO inhibition is a promising target for asthma therapy. The 5-LO redox inhibitor zileuton (Zyflo IR/CR®) is currently marketed for the treatment of asthma in adults and children, but widespread use of zileuton is limited by its efficacy/safety profile, potentially related to its redox characteristics. Thus, a quantitative, mechanistic description of its functioning may be useful for development of improved anti-inflammatory targeting this mechanism.

**Results:**

A mathematical model describing the operation of 5-LO, phospholipase A2, glutathione peroxidase and 5-hydroxyeicosanoid dehydrogenase was developed. The catalytic cycles of the enzymes were reconstructed and kinetic parameters estimated on the basis of available experimental data. The final model describes each stage of cys-leukotriene biosynthesis and the reactions involved in oxoETE production. Regulation of these processes by substrates (phospholipid concentration) and intracellular redox state (concentrations of reduced glutathione, glutathione (GSH), and lipid peroxide) were taken into account. The model enabled us to reveal differences between redox and non-redox 5-LO inhibitors under conditions of oxidative stress. Despite both redox and non-redox inhibitors suppressing leukotriene A4 (LTA4) synthesis, redox inhibitors are predicted to increase oxoETE production, thus compromising efficacy. This phenomena can be explained in terms of the pseudo-peroxidase activity of 5-LO and the ability of lipid peroxides to transform 5-LO into its active form even in the presence of redox inhibitors.

**Conclusions:**

The mathematical model developed described quantitatively different mechanisms of 5-LO inhibition and simulations revealed differences between the potential therapeutic outcomes for these mechanisms.

## Background

Leukotrienes are key inflammatory mediators associated with pathological states of inflammation in diseases such as asthma and allergic rhinitis and play a pivotal role in normal host defense [1]. They have been shown to promote leukocyte chemotaxis and activation, vascular tone and permeability, smooth muscle contractility and immune function. 5-lipoxygenase (5-LO) is the key enzyme of leukotriene biosynthesis and so is a promising target for drug development [2,3].

**Figure 1:**
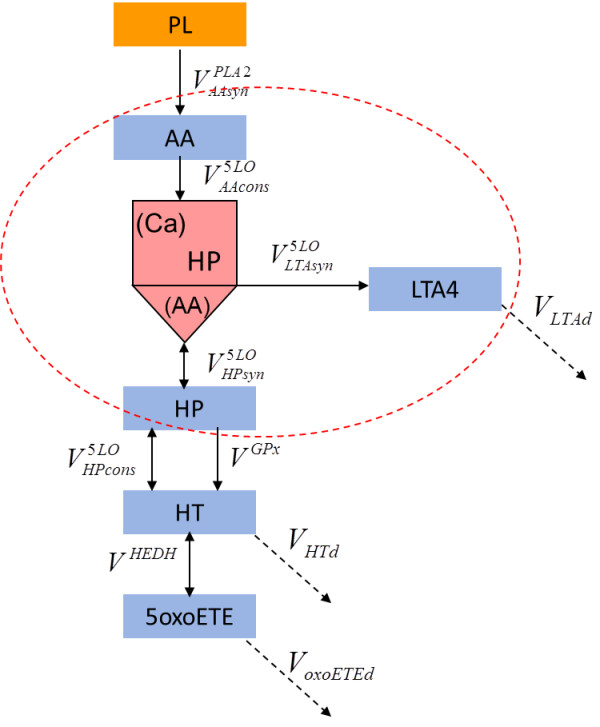
**Schematic representation of Leukotriene and oxoETE synthesis model (“LOS model”).** The reactions occurring with 5-LO are in the dashed red circle. Blue rectangular represents metabolites which are variables of “LOS model”. Pink colour indicates complex of 5-LO and HP. Dashed arrows stand for degradation processes.

5-LO is expressed predominantly in leukocytes and is responsible for the synthesis of both leukotriene A4 (LTA4) and 5(S)-hydroperoxy-6,8,1l,14-(E,Z,Z,Z)-eicosatetraenoic acid (HP) [4-6]. The reaction scheme is given in Figure 1. There are two steps in this reaction: oxygenation of arachidonic acid (AA) using O_2_ to produce HP and the dehydration of the hydroperoxide intermediate, to produce the epoxide, leukotriene A4 (LTA4). HP can be further converted either to 5-hydroxyeicosatetraenoic acid (HT) by glutathione peroxidase (GPx) [7,8]. HT, in turn, can be converted to 5-Oxo-6E,8Z,11Z,14Z-eicosatetraenoic acid (oxoETE) by 5-hydroxyeicosanoid dehydrogenase (HEDH) [9]. oxoETE is produced by various cells including neutrophils, eosinophils, and monocytes [10] and acts as a potent chemo-attractant for these cell types. For example, 5oxoETE stimulates eosinophil migration and tissue infiltration 30 fold more potently than leukotriene B4 (LTB4) [11], and also increases intracellular calcium (Ca^2+^) concentration and actin polymerization in eosinophils [10]. 

5-LO activity is controlled by the intracellular Ca^2+^ concentration and the cellular redox state [4-6,12,13]. The redox state modulates activity via the iron (Fe) atom located in the catalytic site of the enzyme. HP and other lipid peroxides are able to oxidize the Fe atom from Fe^2+^ (ferrous state) to the active Fe^3+^ (ferric state). Consistent with this the addition of glutathione peroxidase (GPx) inhibits formation of products of 5-LO catalyzed reactions *in vitro*[13]. Similar to other lipoxygenases, 5-LO also demonstrates redox state dependent hydroperoxidase activity [14]: ferrous 5-LO reacts with lipid-hydroperoxide to form ferric 5-LO, an oxygen-centered lipid radical and hydroxide ion. Additionally, 5-LO activity, in the presence of Ca^2+^, is increased by structural stabilization via ATP (adenosine-5’-triphosphate) without ATP hydrolysis, microsomal membranes or phosphatidylcholine vesicles (PtdCho) [13]. Mitogen activated protein MAP kinase cascade activity, nuclear import and export of 5-LO, interaction with coactosin-like protein (CLP), 5-LO activating protein (FLAP) and phosphorylation of the enzyme by protein kinase A, resulting in suppression of 5-LO activity, also have been reported to modulate it’s activity [12].

Most of the known inhibitors of the 5-LO act on the basis of a redox-mechanism or by chelation of the Fe atom of 5-LO [3]. Redox inhibitors reduce the Fe atom from active ferric state to the inactive ferrous state. The complexity of 5-LO regulation and the multiple reaction paths suggests that redox and non-redox inhibitors may have qualitatively and quantitatively different effects on the products of 5-LO catalyzed reactions.

Zileuton (Z, N-(l-benzo(b)thien-2-ylethyl)-N-hydroxyurea or Zyflo IR/CR®), is a redox inhibitor of 5-LO currently approved for the daily treatment of asthma in adults and children [15]. Zileuton has a sub-optimal pharmacokinetic and pharmacodynamic profile resulting in a high total daily dose (2400mg) and frequent dosing (4 times a day [q.i.d] for Zyflo IR® and twice a day [b.i.d] for Zyflo CR®) [16], plus a potential for hepatotoxicity [17]. Therefore, the development of a medicine with more convenient dosing regimen may maximise the benefits of inhibiting the leukotriene pathway and provide efficacy superior to that obtained with zileuton. PF-4191834, 4-(3-(4-(1-methyl-1H-pyrazol-5-yl)phenylthio)phenyl)-tetrahydro-2H-pyran-4-carboxamide (PF), is a novel non-iron chelating, non-redox, 5-LO inhibitor under investigation for the treatment of various inflammatory conditions [18]. The presence of significant data in the public domain for zileuton and PF suggested that development of a mathematical model would allow insight into the potential differential effects of non-redox and redox inhibitors. To characterize these potential effects it was necessary to attempt to capture the key properties of the entire pathway and its interactions in inflammatory states Therefore, a detailed mathematical model, describing the processes of 5-LO mediated catalysis regulation (self-inactivation, effect of redox state of the medium) was developed.

Several mathematical models of 5-LO have already been reported, for example a model describing the inhibition of lipoxygenase activity by one of the substrates of the enzyme, AA, by Aharony et al. [19]. In this model, 5-LO is able to bind 2 molecules of AA simultaneously (one molecule in the catalytic site and other molecule in the additional regulatory site) which renders the enzyme catalytically inactive. However, LTA synthesis and the pseudo-peroxidase reaction have not been taken into account in this model. An alternative model of reticulocyte lipoxygenase was developed with 9,12(Z,Z)-octadecadienoic acid (linoleic acid) as substrate [20]. This model did take into account both the activation of the enzyme by product, hydroperoxy derivative of fatty acid, and inhibition of 5-LO with substrate, polienoic fatty acid. However, the substrate inhibition was described in terms of a competitive mechanism, binding to inactive form of 5-LO thus preventing its activation, and the LTA4 synthase activity of the enzyme was not captured. Additionally, none of the models describe the reversible inactivation of 5-LO with HT [21] and irreversible inactivation with LTA4 [22].

The rate laws for lipoxygenase and LTA4 synthase reactions were derived by Yang et al. [23], where the influence of various inhibitors of 5-LO and cyclooxygenase on AA metabolism were determined. These rate equations take into account the inhibition of 5-LO activity by LTA4, HP and HT. However, the inhibition with LTA4 was also described as reversible and the substrate inhibition, product activation and pseudo-peroxidase activity of 5-LO were not taken into account.

Therefore, an opportunity exists to develop a more detailed model of 5-LO activity which describes all the activities of the enzyme and their regulation by substrates and products. The main purpose of this paper is to summarize the development of a detailed mathematical model of 5-LO operation, its application to describe the production of LTA4 and oxoETE, and to study the differences between redox and non-redox inhibitors. The “LOS (Leukotriene-OxoeETE-Synthesis) model” (Figure 1) includes four enzymes: 5-LO, cytosolic phospholipase A2 (cPLA2), GPx and HEDH and describes the major interactions between the components of the system (for example, the influence of glutathione concentration on 5-LO activity). We used the “LOS model” to predict the dose-responses of various inflammatory mediators to redox and non-redox inhibitors and provide a mechanistic explanation for the differences between them.

## Methods

### Model construction

The “LOS model” describing LTA4 and oxoETE production in leukocytes includes reactions catalyzed by 5-LO, cPLA2, GPx, HEDH and degradation of HT, oxoETE and LTA4. Since all leukotrienes are synthesized from LTA4, analysis of 5-LO catalyzed LTA4 production was deemed sufficient to evaluate the impact of 5-LO on leukotriene production. Catalytic cycles for each of the enzymes were constructed and rate equations describing the dependence of reaction rate on concentrations of substrates, products and effectors were derived utilizing literature data.

### The kinetic model of 5-LO

#### Known experimental data and hypotheses used for the model development

In this study, we used the following available experimental data and facts on structural and functional properties of 5-LO:

1. AA inhibits 5-LO activity in lipoxygenase reaction at high concentrations (substrate inhibition) [24].

2. LTA4 can be synthesized from exogenous HP [25].

3. 5-LO is self-inactivating [25]. Glutathione peroxidase and glutathione protect the enzyme from inactivation and lipid peroxides eliminate the protective effect of glutathione.

4. HT is a reversible inhibitor of 5-LO [21].

5. LTA4 can inactivate 5-LO irreversibly [22].

6. The Fe atom in the catalytic site exists in two possible states Fe^2+^ and Fe^3+^. Fe^3+^ is the catalytically active state. Transition between Fe^2+^ and Fe^3+^ states proceeds via oxidation by lipid peroxides, including HP [12]. Reduction from Fe^3+^ to Fe^2+^ state can be mediated by redox inhibitors (zileuton).

7. Oxidation of the enzyme (Fe^2+^ → Fe^3+^ transition) is influenced by Ca^2+^ ions [25].

8. Endogenously generated 5-HP is the preferential substrate for the 5-LO mediated LTA-synthase reaction [26].

9. ATP and membrane binding are necessary for 5-LO activation [13].

During model building and simplification, the following assumptions were made:

a. There are two sites for AA binding: the catalytic site and regulatory site. HT is a competitive inhibitor at the catalytic site.

b. Binding of AA to the regulatory site results in formation of dead-end complexes with 5-LO.

c. Binding of AA and HT to the catalytic site are fast reactions in comparison to product formation.

d. Oxidation and reduction of 5-LO (transition between Fe^2+^ and Fe^3+^) can result from (i) interaction with lipid peroxides or (ii) “spontaneously” (by means of interaction with oxidative or reducing factors: O_2_, H_2_O_2_, thiol groups etc.).

e. Ca^2+^ is able to bind to the catalytic site, but it influences only oxidation and reduction reactions, thus kinetic parameters of other reactions of catalytic cycle remain unchanged.

f. Binding of Ca^2+^ and redox-inhibitors (Z) are fast reactions in comparison to the other reactions of the catalytic cycle.

g. Redox inhibitors are only able to bind to the catalytic site of 5-LO and only when it is not occupied with other factors.

h. The binding of redox inhibitors to the catalytic site blocks the binding of other factors .

i. The regulatory site is able to bind AA only if following conditions are fulfilled: (i) 5-LO is in Fe^3+^ state and (ii) the enzyme is not bound to a redox-inhibitor.

j. The oxygenase, pseudoperoxydase and LTA4 synthase activities of 5-LO were simplified by not describing electron transfer and oxygen binding. Instead, it was assumed that the oxygen concentration is in saturation, i.e., it is not a parameter of the model.

k. To decrease the number of unknown parameters in our model we have assumed that binding of AA to the regulatory site of 5-LO does not influence the binding of AA, HP and HT to the catalytic site (see Additional file 1: Appendix 1).

l. Interaction with MAP kinases, FLAP, CLP and transport of 5-LO to the nucleus have not been taken into account in the model.

#### Catalytic cycle of 5-LO

**Figure 2:**
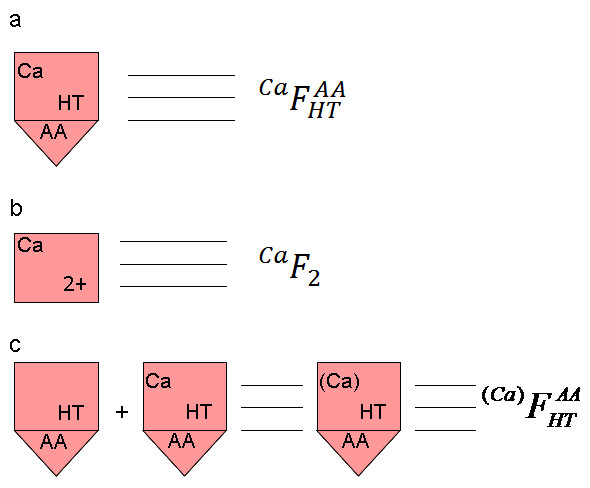
**Examples of notations of 5-LO states.** Notations in kinetic schemes are given at the left and in the text and equations are given at the right: **a**) ferric state of 5-LO with Ca^2+^ and HT bound to catalytic site of the enzyme; **b**) ferrous state with Ca^2+^ bound; **c**) sum of concentrations of ferric state with and without Ca^2+^ bound and with HT and AA bound.

Figure 2 shows schematic representation of the enzyme states considered in the model. In the ferric state 5-LO is able to bind any substrates/products/inhibitors at its catalytic and regulatory sites. This state of 5-LO was represented as a square with a triangle underneath the square. The square designates the catalytic site of the enzyme and the triangle represents the regulatory site. The regulatory site can be found in 2 states: free or AA bound. The catalytic site of 5-LO is able to bind AA, HT, HP, PF (non-redox inhibitor), Z (redox inhibitor). As an example Figure 2(a) shows the HT and AA bound enzyme state. All the above mentioned compounds compete for the substrate binding part of the catalytic site. In addition to the substrate/product (AA, HP) and competitive inhibitors (HT, PF and Z) the catalytic site of 5-LO is able to bind Ca^2+^ (as shown in Figure 2(a)) which does not compete with AA, HT, HP, PF and Z. Therefore, the catalytic site of the ferric enzyme can be found in a total of 12 states: free of any substrate/competitor and with AA, HT, HP, PF, Z bound all of which can be found with and without Ca^2+^ bound. On the basis of this analysis we can conclude that catalytic cycle of 5-LO includes 24 potential states (See Additional file 2: Appendix Figures A1 and A2). The ferrous state (Fe^2+^) of 5-LO is not able to bind any substrate/product/inhibitor at the catalytic or regulatory site but it is able to bind Ca^2+^ at the catalytic site (see Figure 2(b)). Thus, total catalytic cycle includes 26 states of 5-LO. Since in derivation of the rate equations describing 5-LO activities we have used new variables representing sums of states of 5-LO, notations for such sums have also been introduced (Figure 2(c)). In the equations and text of this paper we have used the simplified notations of the 5-LO states (see Figure 1).

Transitions between the states are described in accordance to mass action law and can be either reversible (for example, binding AA to catalytic site) or irreversible (for example, the LTA-synthase reaction). Moreover, these processes can be either relatively fast or slow depending on the values of rate constants obtained from experimental data fitting. This grouping of all processes into two sets (fast and slow processes) allowed us to reduce the initial complexity of the catalytic cycle and derive rate equations describing the operation of 5-LO according to the methods described in [27].

**Figure 3:**
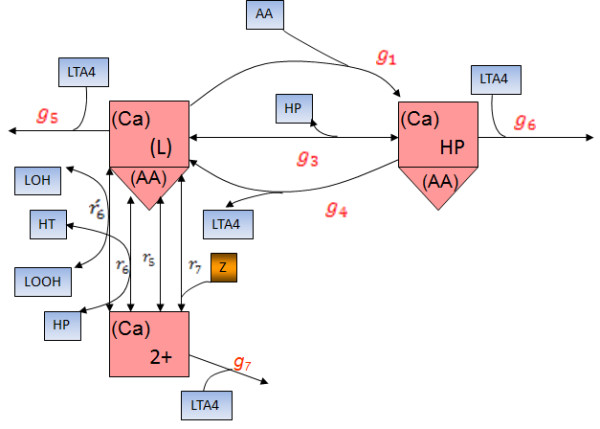
**Reduced catalytic cycle of 5-LO.** Blue rectangular represents metabolites which are variables of “LOS model”. Designation of enzyme states corresponds to Figure 2. Letters with indexes designate effective rate constants (see Additional file 1: Appendix 1).

Schematic visualization of the total catalytic cycle is not convenient because of the complexity (26 nodes/states and tenths of transitions between them). To reconstruct a reduced total catalytic cycle and derive rate equations we employed a step-by-step strategy described in Additional file 1: Appendix 1. As a result we have developed a reduced catalytic cycle (Figure 3) describing oxygenation, dehydration and pseudoperoxidase activities of 5-LO. On the basis of this reduced catalytic cycle, we have derived rate equations (1–7) describing 5-LO mediated AA consumption (*V*_*AAcons*_^5*LO*^), 5-HP production in oxygenase reaction (*V*_*HPcons*_^5*LO*^), HT production and HP consumption in pseudoperoxidase reaction (*V*_*HPcons*_^5*LO*^), all other lipid peroxide (LOOH) consumption in pseudoperoxidase reaction [*V*_*LOOHcons*_^5*LO*^] and LTA4 production (*V*_*LTAsyn*_^5*LO*^):

(1) $$V_{\mathrm{AAcons}}^{5LO}=k_{\mathrm{lo}}\;\frac{AA}{K_{\mathrm{AA}}\Delta_{Z}}\frac{F_{a}}{\Delta_{\mathrm{tot}}}$$

(2) $$V_{\mathrm{LTAsyn}}^{5LO}=k_{\mathrm{LTAsyn}}\;\Delta_{\mathrm{HP}}\frac{Fa}{\Delta_{\mathrm{tot}}\;\delta }$$

(3) $$V_{\mathrm{HPsyn}}^{5LO}=k_{3}\frac{F_{a}}{{{}^{\Delta }}_{\mathrm{tot}}}\;\left(\frac{HP}{\Delta_{Z}K_{d3}}\;\delta_{HP-\Delta_{\mathrm{HP}}}\right)$$

(4) $$V_{\mathrm{HPcons}}^{5LO}=\frac{F_{a}}{{{}^{\Delta }}_{\mathrm{tot}}}\;\rho_{1}\left(HP\;\Delta_{\mathrm{redox}}-\frac{HT}{K_{\mathrm{ox}}}\right)$$

(5) $$V_{\mathrm{HPsyn}}^{5LO}=V_{\mathrm{HPcons}}^{5LO}$$

(6) $$V_{\mathrm{LOOHcons}}^{5LO}=\frac{F_{a}}{{{}^{\Delta }}_{\mathrm{tot}}}\rho_{1}\left(LOOH\;\Delta_{\mathrm{redox}}-\frac{LOH}{K_{\mathrm{ox}}}\right)$$

(7) $$V_{\mathrm{LOHsyn}}^{5LO}=V_{LOOHcons'}^{5LO}$$

Where

(8) $$\Delta_{\mathrm{tot}}=\delta L+\Delta_{\mathrm{HP}}+\Delta_{\mathrm{redox}}$$

(9) $$\Delta HP=\frac{k_{\mathrm{lo}}\;\frac{AA}{k_{\mathrm{LTAsyn}}}+\frac{k_{3}}{k_{d3}}\;HP\;\delta HP}{\Delta_{Z}\;\left(\frac{{}^{k}LTAsyn}{\delta HP}+k_{3}\right)}$$

(10) $$\delta_{L}=1+\frac{AA}{K_{\mathrm{AA}}^{i}{△}_{Z}}+\delta HP\;\left(\frac{AA}{K_{\mathrm{AA}}}+\frac{HT}{K_{\mathrm{HT}}}+\frac{PF}{K_{\mathrm{PF}}}\right)\frac{1}{\Delta_{Z}}$$

(11) $$\delta HP=1+\frac{AA}{{k^{i}}_{\mathrm{AA}}}$$

(12) $$\Delta_{\mathrm{redox}}=\frac{\frac{\rho_{2}}{K_{r}}+\frac{\rho_{1}}{K_{\mathrm{ox}}}*\left(LOH-HT\right)+\rho_{7}*Z}{\left(\rho_{2}+\rho_{1}*\left(LOOH+HP\right)\right)}\frac{{△}_{Z}^{\mathrm{Ca}}}{{△}_{2}^{\mathrm{Ca}}}$$

(13) $$\rho_{1}=k_{\mathrm{ox}}+k_{ox2}\;\frac{Ca}{K_{2}^{\mathrm{Ca}}}\;$$

(14) $$\rho_{2}=k_{r}+k_{r2}\;\frac{Ca}{K_{2}^{\mathrm{Ca}}}\;$$

(15) $$\rho_{7}=k_{\mathrm{ing}}\;\left(1+\frac{Ca}{K_{3}^{\mathrm{Ca}}}\right)\;$$

(16) $$\Delta_{Z}=1+\frac{Z}{Kdz}$$

(17) $${△}_{z}^{\mathrm{Ca}}=1+\frac{Z}{K_{\mathrm{dz}}}+\frac{Ca}{K_{3}^{\mathrm{Ca}}}+\frac{Z}{K_{\mathrm{dz}}}*\frac{Ca}{K_{3}^{\mathrm{Ca}}}=\left(1+\frac{Z}{K_{\mathrm{dz}}}\right)*\left(1+\frac{Ca}{K_{3}^{\mathrm{Ca}}}\right)\;$$

(18) $${△}_{2}^{\mathrm{Ca}}=1+\frac{Ca}{K_{2}^{\mathrm{Ca}}}$$

Since 5-LO undergoes irreversible inactivation, the concentration of active enzyme tends to zero with time and, consequently, all enzyme catalyzed reactions become equal to zero. *In vivo*, regulatory mechanisms control intracellular 5-LO production *de novo*[13]. To obtain non-zero steady state concentrations of 5-LO states and, consequently, to derive the rate equations describing the activities of 5-LO, we did not consider self-inactivation of the enzyme and have not taken into account processes responsible for 5-LO production *de novo*. Under these assumptions the total concentration of active enzyme (*F*_*a*_) is equal to total enzyme concentration and *F*_*a*_ is a parameter of the model. Additionally, identification of model parameters using *in vitro* experimental data was performed on the basis of a model which takes into account self-inactivation of 5-LO with time (see Additional file 1: Appendix 1.5). Under these conditions *F*_*a*_ represents the sum of active states of 5-LO and changes with time. All rate equations were derived on the basis of the quasi-steady state approach [27].

### The kinetic model of phospholipase A_2_

Calcium-dependent phospholipase A2 (cPLA2) catalyzes the production of AA from phospholipids (PL) in the cell membrane. Elevations in the cellular calcium concentration significantly stimulate cPLA2 activity. On the basis of several models of the enzyme developed previously and available experimental data [28,29] we have derived the rate equation for cPLA2 to be (*V*_*AA*_^*PLA*2^, see Additional file 1: Appendix 2):

(19) $$V_{\mathrm{AA}}^{PLA2}=\frac{V_{\max}^{PLA2}\cdot PLA2_{2}^{\mathrm{Ca}}\;PL}{K_{m}^{PLA2_{\mathrm{APC}}}+PL},\;$$

where

(20) $$PLA2_{2}^{\mathrm{Ca}}=\frac{Ca}{K_{\mathrm{Ca}}^{PLA2}+Ca}$$

### The kinetic model of glutathione peroxidase

GPx enzyme reduces HP to HT. This reaction requires glutathione as a cofactor [7,8]. The stoichiometry of the reaction catalyzed by the enzyme is as follows:

(21) HP+2GSH=HT=GSSG

GPx catalyzes this reaction in accordance to the Ping-Pong mechanism and the derivation of the rate equation for GPx is given in Additional file 1: Appendix 3:

(22) $$V^{\mathrm{GPx}}=\frac{GPx_{\mathrm{full}}\cdot B}{A}$$

where

(23) $$A=\frac{HP}{K_{m}^{\mathrm{HP}}}\cdot \left(1+\frac{HT}{K_{\mathrm{HT}}^{\mathrm{GPx}}}\right)+\frac{GSH}{K_{m}^{\mathrm{GSH}}}\cdot \frac{GSH}{K_{m}^{\mathrm{GSH}}}\cdot \left(1+\frac{GSSG}{K_{\mathrm{GSSG}}^{\mathrm{gpx}}}\right)+\frac{GSH}{K_{m}^{\mathrm{GSH}}}\cdot \frac{GSH}{K_{m}^{\mathrm{GSH}}}\cdot \frac{HP}{K_{m}^{\mathrm{HP}}}$$

(24) $$B=\frac{k_{\mathrm{cat}}^{\mathrm{GPx}}\cdot HP}{K_{m}^{\mathrm{HP}}}\frac{GSH}{K_{m}^{\mathrm{GSH}}}\frac{GSH}{K_{m}^{\mathrm{GSH}}}$$

### The kinetic model of 5-hydroxyeicosanoid dehydrogenase

HEDH catalyzes the conversion of HT into oxoETE. The mechanism of HEDH is considered as Bi-Bi Ping-Pong, with NADP as the second substrate [9]. Derivation of the rate equation for HEDH is given in Additional file 1: Appendix 4:

(25) $$V^{\mathrm{HEDH}}=\frac{HEDH_{\mathrm{full}}\;B}{A}\;$$

where

(26) $$B=\frac{k_{1}^{\mathrm{hedh}}\;k_{2}^{\mathrm{hedh}}\;HT\;NADP}{K_{\mathrm{HT}}^{\mathrm{hedh}}\;K_{\mathrm{NADP}}^{\mathrm{hedh}}}-\frac{k_{-1}^{\mathrm{hedh}}\;k_{-2}^{\mathrm{hedh}}\;oxoETE\;NADPH}{K_{\mathrm{oxoETE}}^{\mathrm{hedh}}\;K_{\mathrm{NADPH}}^{\mathrm{hedh}}}$$

(27) $$A=\left(1+\frac{NADP}{K_{\mathrm{NADP}}^{\mathrm{hedh}}}+\frac{oxoETE}{K_{\mathrm{oxoETE}}^{\mathrm{hedh}}}\right)\cdot \left(\frac{k_{1}^{\mathrm{hedh}}\;HT}{K_{\mathrm{HT}}^{\mathrm{hedh}}}+\frac{k_{-2}^{\mathrm{hedh}}\;NADPH}{K_{\mathrm{NADPH}}^{\mathrm{hedh}}}\right)+\left(1+\frac{HT}{K_{\mathrm{HT}}^{\mathrm{hedh}}}+\frac{NADPH}{K_{\mathrm{NADPH}}^{\mathrm{hedh}}}\right)\cdot \left(\frac{k_{-1}^{\mathrm{hedh}}\;oxoETE}{K_{\mathrm{oxoETE}}^{\mathrm{hedh}}}+\frac{k_{2}^{\mathrm{hedh}}\;NADP}{K_{\mathrm{NADP}}^{\mathrm{hedh}}}\right)$$

### The LTA4 and oxoETE synthesis model (“LOS model”)

To build the “LOS model” we utilized the rate equations describing the activities of 5-LO, phospholipase A2, glutathione peroxidase and 5-hydroxyeicosanoid dehydrogenase as given above. The kinetic scheme of the “LOS model” is shown in Figure 1. In accordance with the scheme, AA binding to 5-LO is converted to HP via the lipoxygenase reaction. As a result a complex of 5-LO and HP is formed. HP can be either released from the complex (*V*_*HPsyn*_^5*LO*^) or used to form LTA4 via the LTA4-synthase reaction *V*_*LTAsyn*_^5*LO*^. Additionally, LTA4 can be produced from free HP in the absence of AA (see sequence of reversible reaction *V*_*HPsyn*_^5*LO*^ and irreversible reaction *V*_*LTAsyn*_^5*LO*^). To present all these process correctly the intermediate state of the enzyme ^(*Ca*)^*F*_*HP*_^(*AA*)^ (complex 5-LO with HP) was added to the kinetic scheme. The concentration of the state ^(*Ca*)^*F*_*HP*_^(*AA*)^ is not a variable of the model, i.e. there are no differential equations describing the time dynamics of ^(*Ca*)^*F*_*HP*_^(*AA*)^. However, in accordance with the quasi-steady state approach chosen to describe 5-LO operation in the “LOS model” (and, consequently, applied to derive rate equations of various 5-LO activities) concentration of state ^(*Ca*)^*F*_*HP*_^(*AA*)^ is expressed in terms of variables of the “LOS model” (see Additional file 1: Appendix 1).

To avoid unlimited accumulation of metabolites resulting from constant influx of AA we have introduced processes of degradation of HT, oxoETE and LTA4 (*V*_*HTd*_, *V*_*LTAd*_ and *V*_*oxoETEd*_) in the model. The reaction rates of these processes are described in accordance with mass action law (Additional file 1: Appendix 5). Additionally, concentrations of PL, lipid peroxide LOOH and its reduced product LOH, reduced (GSH) and oxidized (GSSG) glutathione, and reduced and oxidized forms of NADPH are considered as parameters of the model, i.e., do not change with time. The values for the intracellular concentrations of GSH, GSSG, NADPH and NADP were taken from the following sources [30-34]. The concentration of LOOH has either been chosen on the basis of known experimental conditions or has been varied to describe various oxidative states of the cells.

Based on all the above assumptions the system of differential equations describing the “LOS model” is presented below:

(28) $$\frac{dAA}{dt}=V_{\mathrm{AA}}^{PLA2}-V_{\mathrm{AAcons}}^{5LO}\;$$

(29) $$\frac{dHP}{dt}=V_{\mathrm{HPsyn}}^{5LO}-V_{\mathrm{HPcons}}^{5LO}-V^{\mathrm{GPx}}$$

(30) $$\frac{dHT}{dt}=V_{\mathrm{HPcons}}^{5LO}+V^{\mathrm{GPx}}-V^{\mathrm{HEDH}}-V_{\mathrm{HTd}}\;$$

(31) $$\frac{dLTA4}{dt}=V_{\mathrm{LTAsyn}}^{5LO}-V_{\mathrm{LTAd}}\;$$

(32) $$\frac{doxoETE}{dt}=V^{\mathrm{HEDH}}-V_{\mathrm{oxoETEd}}$$

### Description of the parameters of the “LOS model” and experimental data used for their identification

According to assumption L of the section “Known experimental data and hypotheses used for the model development”, some parameters were equated with each other (see 1.4). Thus, for 5-LO 17 independent parameters remained, among them 11 equilibrium constants and 6 rate constants. Additionally, 5 parameters for GPx, and 8 parameters for HEDH needed to be identified. Several of the values of the parameters have been directly taken from other literature sources- e.g. the Michaelis constant for glutathione (*K*_*m*_^*GSH*^) for glutathione peroxidase reaction [7], the rate constant of LTA4 and HT degradation [23]. The values for other parameters were chosen on the basis of the best coincidence between modeling results and corresponding experimental data. To select the values of the parameters we used the algorithm of fitting based on the Hook-Jeeves method [35] implemented in the DBSolve Optimum package [36]. As a criterion of fitness, the following function was used:

(33) $$f\left(k_{j,}K_{j,}\right)=\sum_{i}^{n}{\left(v_{i}-{\bar{v}}_{i}\right)}^{2}$$

Here, *n* is the total number of experimental points, ${}_{i}\bar{v}$ is the experimentally measured value of the variable or reaction rate, *v*_*i*_ is the value of the variable or reaction rate calculated based on the model at a point corresponding to the experimental ones.

Given the complexity of the model, simultaneous identification of parameter estimates would be challenging. Therefore, parameter identification was performed individually for each enzyme by fitting to literature data sets pertinent to the specific enzyme. For example, the parameters of 5-LO were identified via fitting of the 5-LO model against more than 10 experimentally measured curves (76 experimental points) [22,24-26,37,38], 4 unknown parameters of GPx were identified on the basis of 12 experimentally measured points [39] and the parameters of HEDH have been fitted against 47 experimental points [9].

## Results and discussion

### Modeling of 5-LO kinetics

**Figure 4:**
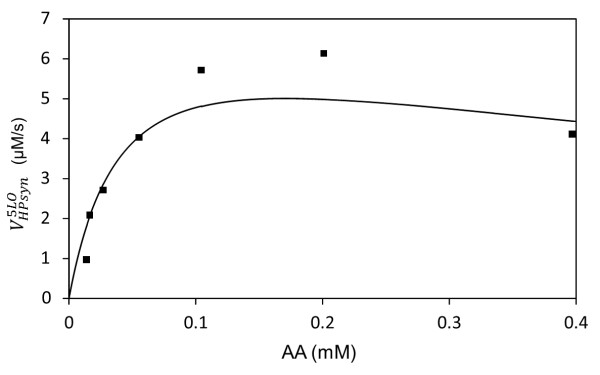
**Dependence of rate of HP synthesis on AA concentration**[24]**.** Experimental conditions: 0.2 mM ATP, 0.3 mM CaCl2. Dots correspond to experimental data; solid line is model generated curve.

**Figure 5:**
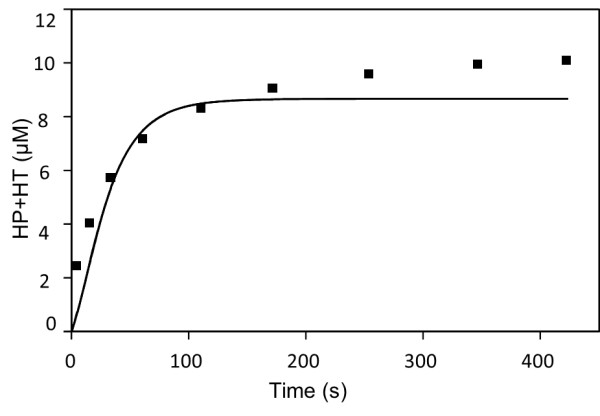
**Time dependence of cumulative concentration of HP and HT.** Experimental conditions: 20 μM of AA, 100 μM of Ca, 0.5 mg of 5-LO in the volume of 500 μL [25]. Dots correspond to experimental data; solid line is model generated curve.

**Figure 6:**
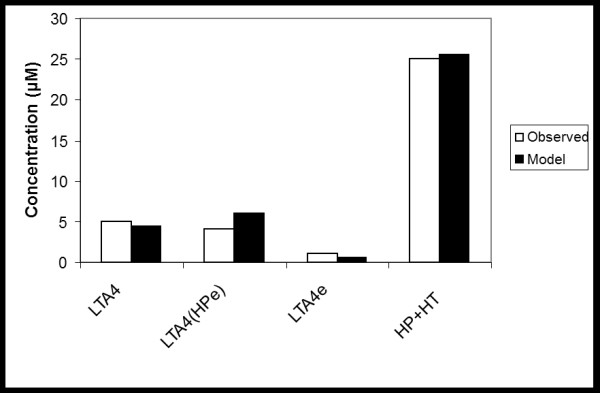
**The effect of exogenous HP on the formation of deuterated LTA4 hydrolysis products.** Human leukocyte homogenate supernatant was incubated with 100 μM octadeuterated arachidonic acid and 80 μM of the exogenous HP, from left to right: LTA4 in the absence of endogenous HP, deuterated LTA4 in presence of exogenous HP, LTA4 formed from exogenous HP, total amount of 5-LO products HP and HT in the absence of exogenous HP.

**Figure 7:**
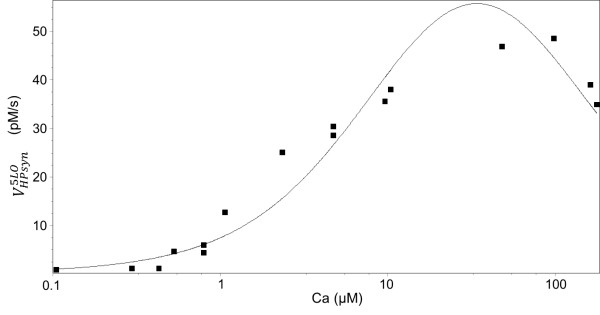
**Dependence of the HP production on the Ca concentration.** Experimental conditions: 20 μM of AA, 0.1 mM ATP. Dots correspond to experimental data from [38], line correspond to results of calculations.

The “LOS model” and parameter values identified during model building enabled us to reproduce various experimental data on the kinetics of 5-LO. Due to the large number of fits only selected representative examples are presented in the main text. The values of the parameters obtained are given in Table 1. Figure 4 demonstrates a model generated curve fitted to the experimentally measured dependencies of HP production rate on AA [24]. Based on this it was concluded that the model of 5-LO satisfactorily described the observed non-monotonic behavior. Figure 5 demonstrates the simulated time series of total concentration of HP and HT fitted to literature data [37]. Additionally, our model satisfactorily fitted experimental data on LTA4 production from endogenous and exogenous HP (see Figure 6) [26] and the Ca^2+^ dependence of 5-HP production by 5-LO on Ca^2+^ concentration [38]. In the latter example the model satisfactorily reproduced both the EC50 value (2–3 μM) and the non-monotonic shape of the experimentally measured dependence (see Figure 7). Other results of fitting are summarized in Additional file 1: Appendix 6. 

**Table 1:** Kinetic parameters of enzyme catalytic cycles

**Parameter**	**Value**	**Units**
*V*_*max*_^*PLA*2^	450	μM/min
*K*_*m*_^*PLA*2 _ *APC*^	20	μM
*K*_*Ca*_^*PLA*2^	0.1	μM
*K*_*AA*_	10.7	μM
*K*_2_^*Ca*^	7.11	mM
*K*_2_^*Ca*^	14.4	μM
*K*_*ox*_	100	μM
*K*_*r*_	5.8 10-7	-
*K*_*AA*_^*i*^	552	μM
*K*_*HT*_	0.54	μM
*K*_*d3*_	1.3 10-4	-
*k*_*lo*_	4.6 103	1/min
*k*_*3*_	0.34 103	(min· μM)^-1^
*k*_*ox*_	2.7 10-4	(min· μM)^-1^
*k*_*ox2*_	67.2	(min· μM)^-1^
*k*_*r*_	2.54 10-4	1/min
*k*_*r2*_	4.4 10-5	1/min
*k*_*LTAsyn*_	5.4 10-4	1/min
*k*_*ing*_	16450	1/min
*K*_*dz*_	36.6	μM
*K*_*PF*_	0.126	uM
*K*_*GSSG*_^*gpx*^	0.072	μM
*k*_*cat*_^*gpx*^	0.489	1/min
*K*_*NADP*_^*hedh*^	2.9	μM
*K*_*NADPH*_^*hedh*^	2.69	μM
*K*_*oxoETE*_^*hedh*^	1.67	μM
*K*_*HT*_^*hedh*^	0.332	μM
*k*_1_^*hedh*^	88.34	1/min
*k*_2_^*hedh*^	1724	1/min
*k*_- 1_^*hedh*^	31.5	1/min
*k*_- 2_^*hedh*^	8.08	1/min
*k*_*HTd*_	0.001	1/min
*k*_*LTAd*_	0.07	1/min
*k*_*oxoETEd*_	0.005	1/min

**Figure 8:**
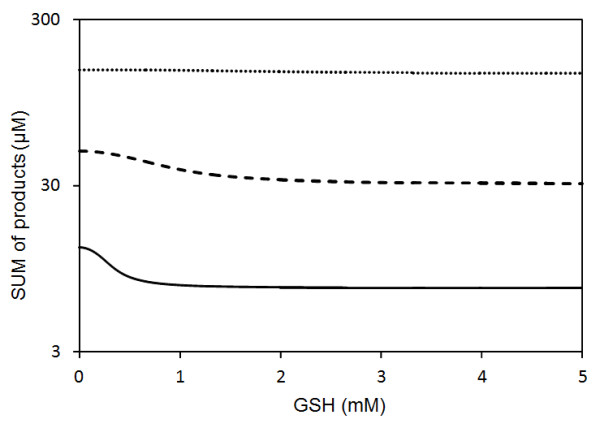
**Simulation of influence of glutathione on production of 5-LO metabolites (sum of LTA4, HP and HT).** Parameters of the LOS model used for simulation: pool of NADP 3 mM; NADPH 2 mM; Ca 1 mM; 5-LO 0.1 μM; HEDH5 0.1 μM; Values of parameter PL responsible for steady state AA level are 117 (solid), 120 (dash), 125 (dot).

The next step of model evaluation examined its ability to reproduce experimentally measured data which had not been used for parameter identification. As an example we selected a dataset describing the influence of glutathione on 5-LO. In accordance with the experimental data [40], the effect of glutathione on 5-LO products in a cell-free extract strongly depends on the concentration of AA. Under conditions of low AA concentrations, the reaction rate of 5-LO is inversely proportional to the glutathione concentration. When AA concentration is high, the rate of 5-LO catalyzed reactions do not decrease even at high concentrations of GSH. Figure 8 demonstrates that our “LOS model” satisfactorily reproduces this threshold influence of GSH on the total concentration of “5-LO metabolites” (sum of HP, HT and LTA4) at various concentrations of AA. Our model explains this phenomenon (threshold like response to GSH increase at various AA concentrations) as driven by the lack of lipid peroxides in this cell-free extract system under conditions of low AA concentrations. In this case the main agent responsible for 5-LO oxidation is HP (see reaction r6 in Figure 3). HP acts as an electron acceptor in the pseudo-peroxidase reaction converting 5-LO from inactive (Fe^2+^) to active (Fe^3+^) state. Under conditions of low AA concentrations, 5-LO is unable to produce a sufficient quantity of HP molecules to drive the transition of the enzyme to active form. Additionally data from experiments with PMN homogenates [40] demonstrated that the addition of GSH can, however, suppress the activation of 5-LO, as long as the AA concentration remains below a critical limit. 

### Modeling of the response to redox and non-redox inhibitors of 5-LO

We applied the “LOS model” to study the influences of redox and non-redox inhibitors on the operation of 5-LO. Inhibitor PF has been profiled in stimulated human whole blood (HWB) against several relevant human targets including 5-LO, 12-LOX, 15-LO and COX enzymes [18]. The compound completely inhibited the synthesis of the 5-LO products (HT, oxoETE, LTB4 and LTE4) with estimated IC50s between 100 and 190 nM. These data demonstrate that the non-redox inhibitor PF is 6–10 times more potent than zileuton.

The mechanism of 5-LO inhibition by the redox and non-redox inhibitors has been modeled under LTA4 steady-state concentration (Additional file 1: Appendix 1). Parameter values describing the kinetic properties of redox and non-redox 5-LO inhibitors were chosen in such a way to provide satisfactory coincidence between the IC50 and IC80 measured experimentally [18] and those calculated by the “LOS model”.

To validate the model describing 5-LO we again utilized comparison of test data sets to simulated outcomes. Examples of such “independent” data sets were

(i) time dependences of HP and HT measured at various zileuton concentrations in *in vitro* experiments with 5-LO [3].

(ii) time dependences of peroxides measured in the presence and absence of PF and zileuton in *ex vivo* experiments [18].

**Figure 9:**
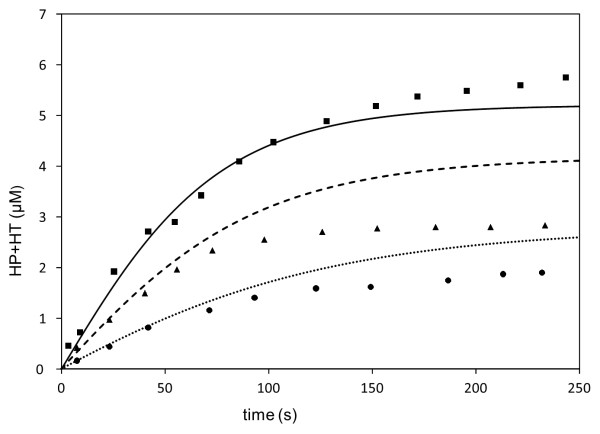
**Dependence of sum of the concentrations of HP and HT on time in presence of zileuton.** Experimental conditions: with no 5-LO inhibitor applied ((squares – data, solid line – model results), with 5 μM of Zileuton applied (triangles – data, dashed line – model results)) and with 20 μM of Zileuton applied (circles – data, dotted line – model results) [3]. Other concentrations: 20 μM of AA, 0.4 mM of Ca.

**Figure 10:**
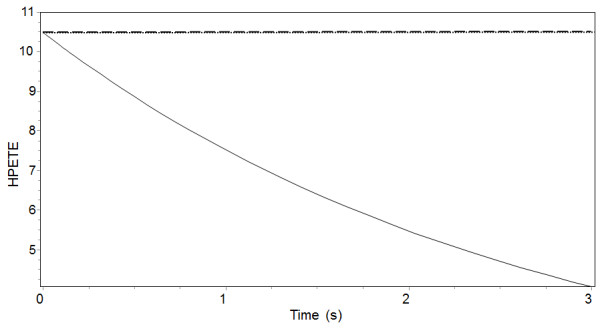
**Quantitative simulation of experiment**[18]**with different inhibitors.** Consumption of 10 μM HP by 5-LO system in presence of 10 μM PF (dotted line) or 10 μM Zileuton (solid line) or in the absence of inhibitors (dashed line, coinciding with dotted line). Concentrations: PL 40 μM, pool of glutathione 10 mM, GSH 5 mM. Other parameters are as in the legend to Figure 8.

Figure 9 demonstrates that the derived values of parameters (Table 1) for zileuton on 5-LO allowed the model to satisfactorily reproduce experimentally measured production of the sum of HP and HT [3]. Moreover, the “LOS model” qualitatively reproduced experimental kinetic data describing the application of redox and non-redox inhibitors in vitro. Experiments in a crude cell lysates 5-LO system containing 10 μM of peroxide (13(S)-HpODE) examined the addition of 10 μM of zileuton or PF (reference). The “LOS model” satisfactory reproduced peroxide consumption following zileuton, and the absence of effect following PF application (Figure 10) due to its non-redox mechanism [18]. 

Thus, comparison of the model simulations to two sets of experimental data which were not used in parameter identification enabled us to conclude that our model adequately described the influence of inhibitors on the system behavior.

### Difference between redox and non-redox inhibitors

As have already been discussed earlier, the redox state of the cell (GSH and lipid peroxide concentration) is able to significantly influence 5-LO activity. In the “LOS model” different levels of oxidative stress were described in terms of LOOH (representing total peroxide concentration except HP). To predict system responses to redox and non-redox inhibitors and to identify possible differences between them at different levels of LOOH we simulated the dependence of steady state LTA4 and oxoETE on PF and zileuton concentration. Several LOOH concentrations were chosen for this study: 0 ÂµM (no oxidative stress), 5 ÂµM (normal level according to available literature data [41]), 10 ÂµM (asthmatic patients [41]) and 100 ÂµM (extremely high oxidative stress). For all simulations the system was run to steady-state conditions with subsequent addition of inhibitor and the effect at the new steady-state noted. This was represented as the percentage of inhibition of LTA4 and oxoETE at a given inhibitor concentration and was calculated using the following equations:

(34) $$Inh_{\%}^{LTA4}=100\;\left(1-\frac{\left[LTA4_{\mathrm{st}}^{\mathrm{inh}}\right]}{\left[LTA4_{\mathrm{st}}^{inh=0}\right]}\right)$$

(35) $$Inh_{\%}^{oxoETE}=100\;\left(1-\frac{\left[oxoETE_{\mathrm{st}}^{\mathrm{inh}}\right]}{\left[oxoETE_{\mathrm{st}}^{inh=0}\right]}\right)\;$$

where *LTA*4_*st*_^*inh*^, *oxoETE*_*st*_^*inh*^,are steady-state concentrations of LTA4 and oxoETE at a specified inhibitor concentration *LTA*4_*st*_^*inh* = 0^, *oxoETE*_*st*_^*inh* = 0^ are steady-state concentrations of LTA4 and oxoETE in the absence of inhibitor.

**Figure 11:**
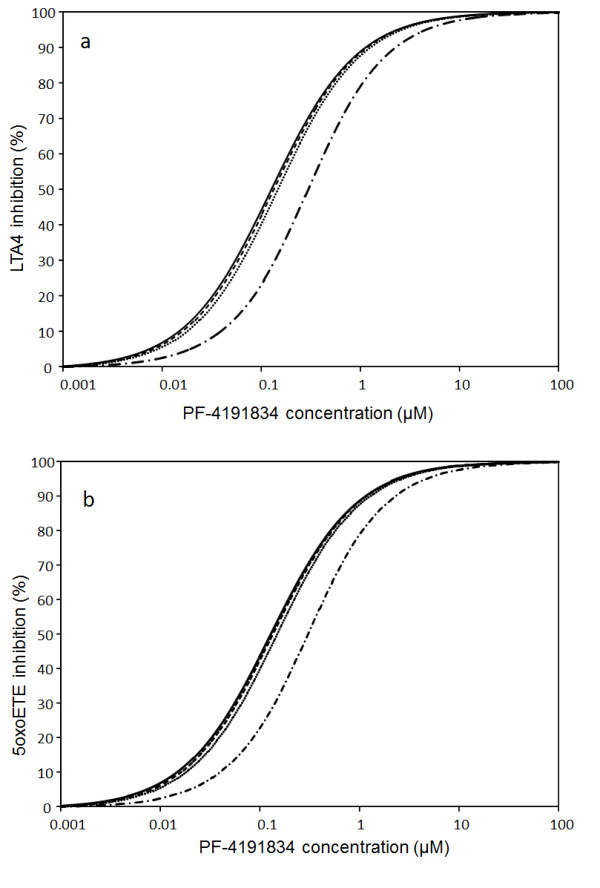
**Simulation of influence of PF on LTA4 and oxoETE production.** Concentrations of lipid peroxide (LOOH): 0 (solid), 5 μM (dash), 10 μM (dot), 100 μM (dash-dot). Other parameters are as in the legend to Figure 10.

**Figure 12:**
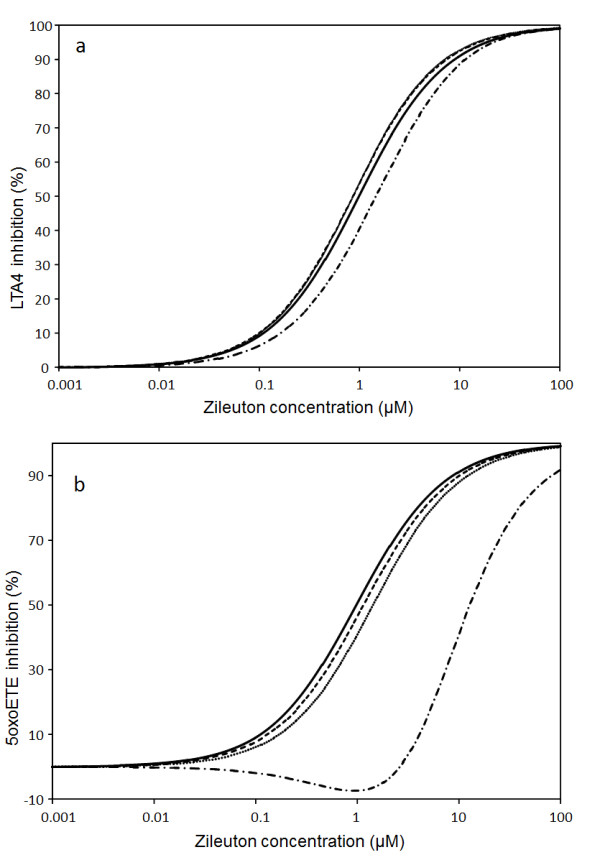
**Simulation of influence of zileuton on LTA4 and oxoETE production.** Concentrations of lipid peroxide (LOOH): 0 (solid), 5 μM (dash), 10 μM (dot), 100 μM (dash-dot). Other parameters are as in the legend to Figure 10.

We have simulated how the LTA4 and oxoETE dose response depends on the LOOH level and found that both were influenced significantly (Table 2). Indeed, assuming LOOH level equal to 0 (no oxidative stress) we have simulated how steady state concentrations of LTA4 and oxoETE depend on zileuton and PF compound concentrations (Figures 11,12, solid lines). Figure 11 demonstrates that model fits satisfactorily *ex vivo* experimental data [18] on the dependence of LTA4 and oxoETE on PF concentration (Tables 3, 2). The solid lines on Figures 11,12 also show that the potency of zileuton to inhibit oxoETE production is the same as for LTA4 production, but it is ten times lower than the potency of PF compound to inhibit both rates (see Tables 3, 2). 

**Table 2:** Potency of 5-LO Inhibitors in “LOS” model at different peroxide concentrations

**Peroxide concentration (μM)**	**IC50 for inhibition by Zileuton (nM)**		**IC50 for inhibition by PF-4191834(nM)**	
	**For LTA4**	**For 5oxoETE**	**For LTA4**	**For 5oxoETE**
0	980	962	122	125
5*	860	1145	125	129
10**	860	1410	143	146
100	1450	12670	290	300

*- corresponds to healthy subjects.

** - corresponds to asthmatic subjects.

**Table 3:** **Potency of 5-LO inhibitors in whole blood from control (Normal) and asthmatic human volunteer**[18]

	**CONTROL**				**ASTHMATICS**			
	**IC50 (nM)**		**IC80 (nM)**		**IC50 (nM)**		**IC80 (nM)**	
	**Experim.**	**Model**	**Experim.**	**Model**	**Experim.**	**Model**	**Experim.**	**Model**
**Zileuton**	850 ± 220	860	2270 ± 710	3330	1080 ± 220	860	2520 ± 1010	3270
**PF-4191834**	120 ± 10	125	300 ± 20	518	180 ± 20	143	390 ± 50	550

Assuming a level of LOOH in healthy controls of 5 ÂµM (see Figures 11– 12, (dashed lines)) we have found that curves describing level of inhibition of LTA4 and oxoETE show a similar tendency but in case of inhibition with zileuton a new feature was observed. The increase in LOOH level from 0 to 5 ÂµM was accompanied by a decrease in zileuton potency for inhibition of oxoETE synthesis (see Figure 12, dashed line) in comparison with its potency to inhibit LTA4 synthesis (IC50_LTA4_<IC50_oxoETE_). This effect is more pronounced when setting the [LOOH] equal to 10 ÂµM (approximating that of asthmatic patients, Figures 11– 12, dotted lines). To further illustrate this effect we simulated the impact of a supra-physiological LOOH concentration of 100 ÂµM on the inhibitory properties of compounds (dash-dot lines in Figures 11b and 12b).

### Discussion of redox-inhibitor properties at high oxidative stress

Zileuton donates an electron to the active (Fe^3+^) state of 5-LO to form the inactive ferrous (Fe^2+^) state and a zileuton radical (see reaction r_7_ in Figure 3). The zileuton radical is eliminated without subsequent reduction to zileuton [42]. To activate 5-LO it is necessary to oxidize the inactive ferrous (2+) state, i.e. an electron donated to an acceptor such as HP or LOOH. Therefore, under conditions of low LOOH only HP is able to oxidize 5-LO. If zileuton is applied, 5-LO is converted to the inactive ferrous state, the rate of HP synthesis decreases and, as a consequence, the enzyme is completely inhibited because of lack of its oxidation. In the absence of other sources of lipid peroxides and HP, 5-LO is completely inhibited by zileuton.

Under conditions of high LOOH, the transformation of ferrous 5-LO state into ferric state can proceed in two possible ways: either via HP or LOOH reduction (see reaction *r*_*6*_ in Figure 3). When zileuton is applied, the concentration of ferrous state of 5-LO increases. However, high level of LOOH may compensate for the lack of HP in the pseudo-peroxidase reaction that leads to maintenance of level of the active 5-LO state and, as a consequence, the lipoxygenase reaction occurs. Under these conditions the rate of pseudo-peroxidase reaction increases and both HP and LOOH are reduced in this reaction converting 5-LO into active ferric state. As a consequence of increased HP consumption in the pseudo-peroxidase reaction, its concentration decreases, leading to a decrease in LTA4 production. At the same time increases in HT concentration produced in the pseudo-peroxidase reaction leads to further increase in oxoETE production.

## Conclusions

We have constructed a model of 5-LO activity and regulation that demonstrates emergent properties consistent with a wide range of experimental data. Utilizing this model we have derived several conclusions:

i) Both redox and non-redox inhibitors of 5-LO decrease LTA4 production independently of lipid peroxide level.

ii) Non-redox inhibitors of 5-LO decrease HT production (and, consequently, 5-oxoETE) independently of lipid peroxide level.

iii) The effect of redox inhibitors on oxoETE production strongly depends on LOOH level;

iv) Redox inhibition of 5-LO under condition of high LOOH switches 5-LO from LTA4 production to HT (and, consequently, oxoETE) production.

OxoETE is one of the key inflammatory mediators and the results of our work presented in the paper have demonstrated that under certain conditions redox inhibitors can lead to a non-intuitive increase in oxoETE synthesis. Together with the PK disadvantages of zileuton this observation supports the further investigation of non-redox inhibitors as possible drugs against asthma.

## Abbreviations

5-LO: 5-Lipoxygenase; AA: Arachidonic acid; HP: 5(S)-Hydroperoxy-6,8,1l,14- (E,Z,Z,Z)-eicosatetraenoic acid; LTA4: Leukotriene A4; HT: 5-Hydroxyeicosatetraenoic acid; PL: Phospholipids; PLA2: Phospholipase A2; GPx: Glutathione peroxidase 1; GSH: Glutathione; HEDH: 5-Hydroxyeicosanoid dehydrogenase; oxoETE: 5-Oxo-6E,8Z,11Z,14Z-eicosatetraenoic acid; PF: 4-(3-(4-(1-Methyl-1H-pyrazol-5-yl)phenylthio)phenyl)-tetrahydro-2H-pyran-4-carboxamide; Z: Zileuton or N-(l-benzo(b)thien-2-ylethyl)-N-hydroxyurea), Zyflo IR/CR®; LOOH: all other lipid peroxides except HP, which can be consumed by 5-LO; LOH: products of pseudoperoxydase reactions with lipid peroxides except HP.

## Competing interests

The authors declare that they have no competing interests.

## Authors’ contributions

TK developed 5LO kinetic model; ODJ developed models for GPx and HEDH; KZ combined the models and described action of inhibitors, simulated inhibition curves; DS described action of inhibitors, simulated inhibition curves; OD, DF and BA took part in study design and combining the models. All authors took part in the conception of the manuscript, drafted and revised the manuscript.

## Supplementary Material

Additional file 1 **Appendix 1.** Derivation of model equations. Click here for file

Additional file 2 **Appendix 2.** Additional figures. Click here for file

## References

[B1] Hammarstrom S Leukotrienes Ann Rev Biochem 1983 52 355 77 10.1146/annurev.bi.52.070183.002035 6311078

[B2] Batt DG 5-lipoxygenase inhibitors and their anti-inflammatory activities Prog Med Chem 1992 29 1 63 1475368 10.1016/s0079-6468(08)70004-3

[B3] Falgueyret J-P Hutchinson JH Riendau D Criteria for the identification of non-redox inhibitors of 5-lipoxygenase Biochem Pharmacol 1993 45 978 981 10.1016/0006-2952(93)90185-Y 8452572

[B4] Ford-Hutchinson AW Gresser M Young RN 5-lipoxygenase Ann Rev Biochem 1994 63 383 417 10.1146/annurev.bi.63.070194.002123 7979243

[B5] Werz O 5-Lipoxygenase: cellular biology and molecular pharmacology Curr Drug Targets Inflamm Allergy 2002 1 23 44 10.2174/1568010023344959 14561204

[B6] Rådmark O Arachidonate 5-lipoxygenase Prostaglandins Other Lipid Mediat 2002 68-69 211 234 12432920 10.1016/s0090-6980(02)00032-1

[B7] Martínez JI García RD Galarza AM The kinetic mechanism of glutathione peroxidase from human platelets Thromb Res 1982 27 2 197 203 10.1016/0049-3848(82)90199-2 7135354

[B8] Chiu DT Stults FH Tappel AL Purification and properties of rat lung soluble glutathione peroxidase Biochim Biophys Acta 1976 445 558 66 10.1016/0005-2744(76)90110-8 974099

[B9] Erlemann KR Cossette C Grant GE Lee GJ Patel P Rokach J Powell WS Regulation of 5-hydroxyeicosanoid dehydrogenase activity in monocytic cells Biochem J 2007 403 157 65 10.1042/BJ20061617 17166093 PMC1828885

[B10] Powell WS Rokach J Biochemistry, biology and chemistry of the 5-lipoxygenase product 5-oxo-ETE Prog Lipid Res 2005 44 154 83 10.1016/j.plipres.2005.04.002 15893379

[B11] Powell WS Chung D Gravel S 5-Oxo-6,8,11,14-eicosatetraenoic acid is a potent stimulator of human eosinophil migration J Immunol 1995 154 4123 32 7706749

[B12] Radmark O Werz O Steinhilber D Samuelsson B 5-Lipoxygenase: regulation of expression and enzyme activity Trends Biochem Sci 2007 32 332 341 10.1016/j.tibs.2007.06.002 17576065

[B13] Radmark O Samuelsson B Regulation of 5-lipoxygenase enzyme activity Biochem Biophys Res Commun 2005 338 102 110 10.1016/j.bbrc.2005.08.013 16122704

[B14] Riendeau D Falgueyret JP Guay J Ueda N Yamamoto S Pseudoperoxidase activity of 5-lipoxygenase stimulated by potent benzofuranol and N-hydroxyurea inhibitors of the lipoxygenase reaction Biochem J 1991 274 287 92 2001245 10.1042/bj2740287 PMC1149951

[B15] Awni WM Locke C Dube LM Cavanaugh JH Evaluation of the diurnal variation in the pharmacokinetics of zileuton in healthy volunteers J Clin Pharmacol 1997 37 388 9156371 10.1002/j.1552-4604.1997.tb04316.x

[B16] Awni WM Braeckman RA Granneman GR Witt G Dubé LM Pharmacokinetics and pharmacodynamics of zileuton after oral administration of single and multiple dose regimens of zileuton 600 mg in healthy volunteers Clin Pharmacokinet 1995 29 22 3 10.2165/00003088-199500292-00005 8620668

[B17] Joshi EM Heasley BH Chordia MD Macdonald TL In vitro metabolism of 2-acetylbenzothiophene: relevance to zileuton hepatotoxicity Chem Res Toxicol 2004 17 137 43 10.1021/tx0341409 14967000

[B18] Masferrer JL Zweifel BS Hardy M Anderson GD Dufield D Cortes-Burgos L Pufahl RA Graneto M Pharmacology of 4-(3-(4-(1-methyl-1H-pyrazol-5-yl) phenylthio) phenyl)-tetrahydro-2H-pyran-4-carboxamide (PF-4191834), a Novel Selective, non-redox, 5-Lipoxygenase Inhibitor Effective in Inflammation and Pain J Pharmacol Exp Ther 2010 334 294 301 10.1124/jpet.110.166967 20378715

[B19] Aharony D Ross L Stein RL Kinetic mechanism of guinea Pig neutrophil 5-lipoxygenase J Biol Chem 1986 261 11512 11519 3091590

[B20] Ludwig P Holzhutter H-G Colosimo A Silvestrini MC Schewe T Rapoport SM A kinetic model for lipoxygenases based on experimental data with the lipoxygenase of reticulocytes Eur J Biochem 1987 168 325 337 10.1111/j.1432-1033.1987.tb13424.x 3117544

[B21] Aharony D Redkar-Brown DG Hubbs SJ Stein RL Kinetic studies on the inactivation of 5-lipoxygenase by 5(S)-hydroperoxyeicosatetraenoic acid Prostaglandins 1987 33 85 100 10.1016/0090-6980(87)90307-8 3108961

[B22] Lepley RA Fitzpatrick FA Irreversible inactivation of 5-lipoxygenase by leukotriene A4. Characterization of product inactivation with purified enzyme and intact leukocytes J Biol Chem 1994 269 2627 2631 8300592

[B23] Yang K Ma W Liang H Ouyang Q Tang C Lai L Dynamic simulations on the arachidonic acid metabolic network PLoS Comput Biol 2007 3 e55 10.1371/journal.pcbi.0030055 17381237 PMC1829479

[B24] Pande AH Moe D Nemec KN Qin S Tan S Tatulian SA Modulation of human 5-lipoxygenase activity by membrane lipids Biochemistry 2004 43 14653 14666 10.1021/bi048775y 15544336

[B25] De Carolis E Denis D Riendeau D Oxidative inactivation of human 5-lipoxygenase in phosphatidylcholine vesicles Eur J Biochem 1996 235 416 423 10.1111/j.1432-1033.1996.00416.x 8631361

[B26] Puustinen T Scheffer MM Samuelsson B Endogenously generated 5-hydroperoxyeicosatetraenoic acid is the preferred substrate for human leukocyte leukotriene A4 synthase activity FEBS Lett 1987 217 265 268 10.1016/0014-5793(87)80675-0 3036580

[B27] Mogilevskaya E Bagrova N Plyusnina T Gizzatkulov N Metelkin E Goryacheva E Smirnov S Kosinsky Y Dorodnov A Peskov K Karelina T Goryanin I Demin O Kinetic modeling as a tool to integrate multilevel dynamic experimental data Methods Mol Biol 2009 563 197 218 10.1007/978-1-60761-175-2_11 19597787

[B28] Kramer RM Roberts EF Manetta JV Hyslop PA Jakubowski JA Thrombin-induced phosphorylation and activation of Ca(2+)-sensitive cytosolic phospholipase A2 in human platelets J Biol Chem 1993 268 26796 26804 8253817

[B29] Lukas TJ A signal transduction pathway model prototype I: From agonist to cellular endpoint Biophys J 2004 87 1406 1416 10.1529/biophysj.103.035253 15345523 PMC1304549

[B30] Smith CV Jones DP Guenthner TM Lash LH Lauterburg BH Compartmentation of glutathione: implications for the study of toxicity and disease Toxicol Appl Pharmacol 1996 140 1 12 10.1006/taap.1996.0191 8806864

[B31] Bruynzeel PL Kok PT Viëtor RJ Verhagen J On the optimal conditions of LTC4 formation by human eosinophils in vitro Prostaglandins Leukot Med 1985 20 11 22 10.1016/0262-1746(85)90090-3 3934683

[B32] Linden M Håkansson L Ohlsson K Sjödin K Tegner H Tunek A Venge P Glutathione in bronchoalveolar lavage fluid from smokers is related to humoral markers of inflammatory cell activity Inflammation 1989 13 651 658 10.1007/BF00914309 2613293

[B33] Graham FD Erlemann KR Gravel S Rokach J Powell WS Oxidative stress-induced changes in pyridine nucleotides and chemoattractant 5-lipoxygenase products in aging neutrophils Free Radic Biol Med 2009 47 62 71 10.1016/j.freeradbiomed.2009.04.016 19376220 PMC2891157

[B34] Sander BJ Oelshlegel FJ Jr Brewer GJ Quantitative analysis of pyridine nucleotides in red blood cells: a single-step extraction procedure Anal Biochem 1976 71 29 36 10.1016/0003-2697(76)90006-3 5911

[B35] Hook R Jeeves TA Direct search solution of numerical and statistical problems J ACM 1961 8 212 229 10.1145/321062.321069

[B36] Gizzatkulov NM Goryanin II Metelkin EA Mogilevskaya EA Peskov KV Demin OV DBSolve Optimum: a software package for kinetic modeling which allows dynamic visualization of simulation results BMC Syst Biol 2010 4 109 10.1186/1752-0509-4-109 20698988 PMC2925829

[B37] Rakonjac M Fischer L Provost P Werz O Steinhilber D Samuelsson B Rådmark O Coactosin-like protein supports 5-lipoxygenase enzyme activity and up-regulates leukotrienee A4 production Proc Nat Acad Sci USA 2006 103 13150 13155 10.1073/pnas.0605150103 16924104 PMC1559768

[B38] Percival D Denis D Riendeau D Gresser MJ Investigation of the mechanism of non-turnover-dependent inactivation of purified human 5-lipoxygenase Eur J Biochem 1992 210 109 117 10.1111/j.1432-1033.1992.tb17397.x 1446663

[B39] Jakobsson PJ Mancini JA Riendeau D Ford-Hutchinson AW Identification and characterization of a novel microsomal enzyme with glutathione-dependent transferase and peroxidase activities J Biol Chem 1997 272 36 22934 9 10.1074/jbc.272.36.22934 9278457

[B40] Hatzelmann A Schatz M Ullrich V Involvement of glutathione peroxidase activity in the stimulation of 5-lipoxygenase activity by glutathione-depleting agents in human polymorphonuclear leukocytes Eur J Biochem 1989 180 527 533 10.1111/j.1432-1033.1989.tb14678.x 2496978

[B41] Cakmak A Zeyrek D Atas A Selek S Erel O Oxidative status and paraoxonase activity in children with asthma Clin Invest Med 2009 32 E327 E334 19796573 10.25011/cim.v32i5.6920

[B42] Charnulitrat W Mason RP Riendeau D Nitroxide metabolites from alkylhydroxylamines and N-hydroxyurea derivatives resulting from reductive inhibition of soybean lipoxygenase J Biol Chem 1992 267 9574 9579 1315759

