# Supplementary material for: Regulation of leukotriene and 5oxoETE synthesis and the effect of 5-lipoxygenase inhibitors: a mathematical modeling approach
Source: BMC Syst Biol. 2012 Nov 12;6:141. doi: 10.1186/1752-0509-6-141 (PMC3546923; doi:10.1186/1752-0509-6-141)
Supplement: Additional file 1 — Appendix 1. Derivation of model equations. [file 1752-0509-6-141-S1.pdf]

Appendix 1. Kinetic model of 5-lipoxygenase.

In appendix the steps of 5LO model building are represented. Schematic visualization of total catalytic cycle is not convenient because of huge complexity (26 nodes/states and tenths of transitions between them). So, we employ following step-by-step strategy to reconstruct and reduce total catalytic cycle and derive rate equations:

(1) To reconstruct "incomplete" catalytic cycle of 5LO which includes **(i)** binding of Ca2+ at catalytic site, **(ii)** Fe2+ → Fe3+ transition, **(iii)** binding of redox inhibitor Z and **(iv)** binding and transformation of AA at catalytic site. It means that binding of AA at regulatory site and binding of HETE and PF at catalytic site have been omitted. "Incomplete" catalytic cycle is presented in Fig. A1.

(2) To reduce this "incomplete" catalytic cycle partially on the basis of available experimental data and assumptions on fast and slow processes, according to technics described elsewhere [28]. In the reduced catalytic cycle, we substituted the initial variables (the enzymatic states in Fig.A1) for new ones which represented the sums of the concentrations of the 5LO forms with and without Ca bound. As a result of the stage we have come to "partially reduced incomplete" catalytic cycle (scheme selected by green dashed circle in Fig A2). All the transitions between these new states are described by rate equations which are the sums of the rates of elementary reactions of the non-reduced catalytic cycle (see Appendix 1.1 for details). The concentration of every enzyme state presented in Fig. A1 can be expressed via the new variables shown on Fig. A2.

(3) To supplement this "partially reduced incomplete" catalytic cycle with all remaining states and transitions which have been omitted at the stage (1). This can be done in such a way to add all processes which are not described in "incomplete" catalytic cycle and, at the same time, to take into account all reductions/simplifications made at stage (2). As a result of the stage we will have "partially reduced" catalytic cycle of 5LOX (Fig. A2), the part of which is "partially reduced incomplete" catalytic cycle (Fig.A2 in green dashed line).

(4) To reduce this "partially reduced" catalytic cycle on the basis of available experimental data and assumptions on fast and slow processes. As a result of the stage we have come to "reduced" catalytic cycle (Fig. 3 of the main text).

(5) To derive rate equations describing 5LO mediated AA consumption (V_{AAcons}^{5LO}), HP production in oxygenase reaction (V_{HPsyn}^{5LO}), HT production and HP consumption in pseudoperoxidase reaction (V_{HPcons}^{5LO}) and LTA4 production (V_{LTAsyn}^{5LO}) on the basis of the "reduced" catalytic cycle.

Appendix 1.1 Description and reduction of "incomplete" cycle.

Fig. A1 represents scheme of "incomplete" catalytic cycle of 5LO which includes (i) binding of Ca2+ at catalytic site, (ii) Fe^{2+} → Fe^{3+} transition, (iii) binding of redox inhibitor Z and (iv) binding and transformation of AA at catalytic site. Here, designations ${}^{X}F_{Y}$, X∈{∅, Ca}, Y∈{2, 3, AA, HP, Z} are explained in Fig. 1 of main text and stand for states of 5LO. X describes if the Ca2+ bound to 5LO (X=Ca) or not (X=∅). Y describes state of catalytic site of enzyme: "2" and "3" mean that Fe located in active site of the 5LO is either in reduced (Fe2+) or oxidized (Fe^{3+}) state; AA, HP and Z mean that arachidonic acid, HP or Zileuton are bound to catalytic site. System of ordinary differential equations corresponding to the scheme presented below:

$$
\begin{aligned}
&\frac{dF_2}{dt} = V_5 + V_8 - V_6 - V_{25} - V_{29} \\
&\frac{d^{Ca}F_2}{dt} = V_{13} + V_{15} - V_{14} - V_{26} + V_{29} \\
&\frac{dF_3}{dt} = -V_1 + V_4 - V_3 - V_5 - V_7 + V_6 - V_{18} - V_{28} \\
&\frac{dF_{AA}}{dt} = V_1 - V_2 - V_{17} - V_{27} \\
&\frac{dF_{HP}}{dt} = V_2 - V_4 + V_3 - V_{19} - V_{30} \\
&\frac{dF_Z}{dt} = V_7 - V_8 - V_{20} - V_{31} \\
&\frac{d^{Ca}F_3}{dt} = -V_9 - V_{13} + V_{14} - V_{16} - V_{12} + V_{11} - V_{22} + V_{28} \qquad (1.1) \\
&\frac{d^{Ca}F_{AA}}{dt} = V_9 - V_{10} - V_{21} + V_{27} \\
&\frac{d^{Ca}F_{HP}}{dt} = V_{12} - V_{11} + V_{10} - V_{23} + V_{30} \\
&\frac{d^{Ca}F_Z}{dt} = V_{16} - V_{15} - V_{24} + V_{31}
\end{aligned}
$$

V_i, i=1,…31, designate rate equations for transitions which are described in accordance to reversible/irreversible mass action law:

$$V_1 = k_{AA} \cdot W_1 = k_{AA} \cdot (F_3 \cdot \frac{AA}{K_{AA}} - F_{AA})$$

$$V_2 = k_{lo} \cdot F_{AA}$$

$$V_3 = k_3 \cdot W_4 = k_3 \cdot \left(\frac{F_3 \cdot HP}{K_{d3}} - F_{HP}\right)$$

$$V_4 = \ k_{LTA\_syn} \cdot F_{HP}$$

$$V_5 = k_r \cdot W_5 = k_r \cdot (F_2 - \frac{F_3}{K_r})$$

$$V_6 = k_{ox} \cdot W_6 = k_{ox} \cdot \left(HP \cdot F_2 - \frac{HT}{K_{ox}} \cdot F_3\right)$$

$$\acute{V}_6 = k_{ox} \cdot W_6 = k_{ox} \cdot (LOOH \cdot F_2 - \frac{LOH}{K_{ox}} \cdot F_3)$$

$$V_7 = k_7 \cdot W_7 = k_7 \cdot (F_3 \cdot \frac{Z}{K_{dZ}} - F_Z)$$

$$V_8 = k_Z \cdot F_Z$$

$$V_9 = k_{AA} \cdot W_9 = k_{AA} \cdot ({}^{Ca}F_3 \cdot \frac{AA}{K_{AA}} - {}^{Ca}F_{AA})$$

$$V_{10} = k_{lo} \cdot {}^{Ca}F_{AA}$$

$$V_{11} = k_{LTA\_syn} \cdot F_{HP}^{Ca}$$

$$V_{12} = k_3 \cdot W_{12} = k_3 \cdot \left({}^{Ca}F_3 * \frac{HP}{K_{d3}} - {}^{Ca}F_{HP}\right)$$

$$V_{13} = k_{r2} \cdot ({}^{Ca}F_2 - \frac{{}^{Ca}F_3}{K_{r2}})$$

$$V_{14} = k_{OX2} \cdot W_{14} = k_{OX2} \cdot (HP \cdot {}^{Ca}F_2 - \frac{HT}{K_{OX2}} \cdot {}^{Ca}F_3)$$

$$\acute{V}_{14} = k_{OX2} \cdot W_{14} = k_{OX2} \cdot (LOOH \cdot {}^{Ca}F_2 - \frac{LOH}{K_{OX2}} \cdot {}^{Ca}F_3)$$

$$V_{15} = k_Z \cdot {}^{Ca}F_Z$$

$$V_{16} = k_{16} \cdot W_{16} = k_{16} \cdot \left({}^{Ca}F_3 \cdot \frac{Z}{K_{dz}} - {}^{Ca}F_Z\right)$$

$$V_{17} = k_{LTAin} \cdot LTA4 \cdot F_{AA} \qquad (1.2)$$

$$V_{18} = k_{LTAin} \cdot LTA4 \cdot F_3$$

$$V_{19} = k_{LTAin} \cdot LTA4 \cdot F_{HP}$$

$$V_{20} = k_{LTAin} \cdot LTA4 \cdot F_z$$

$$V_{21} = k_{LTAin} \cdot LTA4 \cdot {}^{Ca}F_{AA}$$

$$V_{22} = k_{LTAin} \cdot LTA4 \cdot {}^{Ca}F_3$$

$$V_{23} = k_{LTAin} \cdot LTA4 \cdot {}^{Ca}F_{HP}$$

$$V_{24} = k_{LTAin} \cdot LTA4 \cdot {}^{Ca}F_Z$$

$$V_{25} = k_{LTAin} \cdot LTA4 \cdot F_2$$

$$V_{26} = k_{LTAin} \cdot LTA4 \cdot {}^{Ca}F_2$$

$$V_{27} = k_{27} \cdot W_{27} = k_{27} \cdot \left(F_{AA} \cdot \frac{Ca}{K_3^{Ca}} - {}^{Ca}F_{AA} \right)$$

$$V_{28} = k_{28} \cdot W_{28} = k_{28} \cdot \left(F_3 \cdot \frac{Ca}{K_3^{Ca}} - {}^{Ca}F_3 \right)$$

$$V_{29} = k_{29} \cdot W_{29} = k_{29} \cdot \left(F_2 \cdot \frac{Ca}{K_2^{Ca}} - {}^{Ca}F_2 \right)$$

$$V_{30} = k_{30} \cdot W_{30} = k_{30} \cdot \left(F_{HP} \cdot \frac{Ca}{K_3^{Ca}} - {}^{Ca}F_{HP} \right)$$

$$V_{31} = k_{31} \cdot W_{31} = k_{31} \cdot (F_Z \cdot Ca \frac{1}{K_3^{Ca}} - {}^{Ca}F_Z)$$

In accordance with available experimental facts and assumptions described above we can consider processes of Ca^{2+} binding (V_i, i=27,…,31) and redox inhibitor (Z) binding (V_i, i=7, 16) to catalytic site of 5LO as fast processes. Applying technique described elsewhere [28] division of all processes into fast and slow groups allows us to reduce systems of differential equations (1.1) assuming that ki, i=7,16,27,…,31 are much larger than other rate constants. Indeed, summing up equations of the system (1.1) in such a way to exclude "fast" rates from right hand sides of as much differential equations as possible and tending ki, i=7,16,27,…,31 to infinity we transform ODE system (1.1) to following reduced system of algebra differential equations:

$$e_1)\ \frac{d^{(Ca)}F_2}{dt} = (V_5 + V_{13}) + (V_8 + V_{15}) - (V_6 + V_{14}) - (V_{25} + V_{26})$$

$$e_2)\ W_{29} = 0$$

$$e_3)\ \frac{d^{(Ca)}F_{(Z)}}{dt} = -(V_1 + V_9) - (V_5 + V_{13}) + (V_{14} + V_6) - (V_4 + V_{12}) + (V_3 + V_{11}) - (V_{18} + V_{22}) - (V_8 + V_{15}) - (V_{20} + V_{24})$$

$$e_4)\ W_7 = W_{16} = W_{28} = W_{31} = 0$$

$$e_5)\ \frac{d^{(Ca)}F_{AA}}{dt} = (V_1 + V_9) - (V_2 + V_{10}) - (V_{17} + V_{21}) \qquad (1.3)$$

$$e_6)\ W_{27} = 0$$

$$e_7)\ \frac{d^{(Ca)}F_{HP}}{dt} = (V_2 + V_{10}) + (V_4 + V_{12}) - (V_3 + V_{11}) - (V_{19} + V_{23})$$

$$e_8)\ W_{30} = 0$$

Here, ${}^{(Ca)}F_2 {}^{(Ca)}F_Z$, ${}^{(Ca)}F_{AA}$, ${}^{(Ca)}F_{HP}$ are new variables of the ODE system designating sums of states of the enzyme which are interconnected by fast processes in catalytic cycle (see Fig 3):

$$e_1 + e_2 \rightarrow e_1$$

$$e_3 + e_7 + e_6 + e_{10} \rightarrow e_3 \qquad (1.4)$$

$e_4 + e_8 \rightarrow e_4$

$e_5 + e_9 \rightarrow e_5$

$F_2 + {}^{Ca}F_2 = {}^{(Ca)}F_3$

$$F_3 + F_z + {}^{Ca}F_3 + {}^{Ca}F_Z = {}^{(Ca)}F_{(Z)} \qquad (1.5)$$

$F_{AA} + {}^{Ca}F_{AA} = {}^{(Ca)}F_{(AA)}$

$F_{HP} + {}^{Ca}F_{HP} = {}^{(Ca)}F_{(HP)}$

Using equations (1.5) and algebraic equations of system (1.3) we can express all old variables (appearing in system (1.1)) in terms of new variables (appearing in differential equations of system (1.3)):

$$F_2 = \frac{{}^{(Ca)}F_2}{1 + \frac{Ca}{K_2^{Ca}}}$$

$$ {}^{Ca}F_2 = \frac{Ca}{K_2^{Ca}} * \frac{{}^{(Ca)}F_2}{1 + \frac{Ca}{K_2^{Ca}}} \qquad (1.6)$$

$$F_3 = \frac{{}^{(Ca)}F_{(Z)}}{\Delta_z^{Ca}}$$

$$\Delta_z^{Ca} = 1 + \frac{Z}{K_{dz}} + \frac{Ca}{K_3^{Ca}} + \frac{Z}{K_{dz}} * \frac{Ca}{K_3^{Ca}} = \left(1 + \frac{Z}{K_{dz}}\right) * (1 + \frac{Ca}{K_3^{Ca}})$$

$$F_z = \frac{Z}{K_{dz}} * \frac{{}^{(Ca)}F_{(Z)}}{\Delta_z^{Ca}} \qquad (1.7)$$

$$ {}^{Ca}F_3 = \frac{Ca}{K_3^{Ca}} * \frac{{}^{(Ca)}F_{(Z)}}{\Delta_z^{Ca}}$$

$$ {}^{Ca}F_Z = \frac{\frac{Z}{K_{dz}} * \frac{Ca}{K_3^{Ca}} * {}^{(Ca)}F_{(Z)}}{\Delta_z^{Ca}}$$

$$F_{AA} = \frac{{}^{(Ca)}F_{AA}}{\Delta_3^{Ca}}$$

$$F_{AA}^{Ca} = \frac{Ca}{K_3^{Ca}} * \frac{{}^{(Ca)}F_{AA}}{\Delta_3^{Ca}}$$

$$\Delta_3^{Ca} = 1 + \frac{Ca}{K_3^{Ca}}$$

$$F_{HP} = \frac{{}^{(Ca)}F_{HP}}{\Delta_3^{Ca}} \qquad (1.8)$$

$$F_{HP}^{Ca} = \frac{Ca}{K_3^{Ca}} * \frac{{}^{(Ca)}F_{HP}}{\Delta_3^{Ca}}$$

There are several detail balance relationships in catalytic cycle which impose interrelations between several equilibrium dissociation constants

$$K_3^{Ca} \cdot K_{r2} = K_2^{Ca} \cdot K_r$$

$$K_3^{Ca} \cdot K_{OX2} = K_2^{Ca} \cdot K_{OX} \qquad (1.9)$$

Applying these relationships and taking into account how equilibrium dissociation constants can expressed in terms of forward and backward rate constants we have derived following relationships:

$$\left(\frac{k_r}{K_r} + \frac{k_{r2}}{K_{r2}} \cdot \frac{Ca}{K_{d3}^{Ca}}\right) = \left(k_r + k_{r2} \cdot \frac{Ca}{K_{d2}^{Ca}}\right)\frac{1}{K_r}$$

$$\left(\frac{k_{OX}}{K_{OX}} + \frac{k_{OX2}}{K_{OX2}} \cdot \frac{Ca}{K_{d3}^{Ca}}\right) = \left(k_{OX} + k_{OX2} \cdot \frac{Ca}{K_{d2}^{Ca}}\right)\frac{1}{K_{OX}} \qquad (1.10)$$

Introducing designations (ρ_i, i=1,2,7) of following expressions

$$\rho_1 = k_{ox} + k_{ox2} \cdot \frac{Ca}{K_2^{Ca}}$$

$$\rho_2 = k_r + k_{r2} \cdot \frac{Ca}{K_2^{Ca}} \qquad (1.11)$$

$$\rho_7 = k_{ing} \cdot (1 + \frac{Ca}{K_3^{Ca}})$$

slow reaction rates appearing in right hand sides of differential equations of system (1.3) can be grouped and rewritten in terms of the new variables in a following way:

$$r_1 = V_1 + V_9 = k_{AA} \cdot (\frac{AA}{K^{AA}} \cdot \frac{1}{\Delta_z} \cdot {}^{(Ca)}F_{(Z)} - {}^{(Ca)}F_{AA})$$

$$r_2 = V_2 + V_{10} = k_{lo} \cdot {}^{(Ca)}F_{AA}$$

$$r_3 = V_3 + V_{11} = k_3 \cdot \left(\frac{HPETE}{K_{d3}} \cdot \frac{1}{\Delta_z} \cdot {}^{(Ca)}F_{(Z)} - {}^{(Ca)}F_{HP}\right)$$

$$r_4 = V_4 + V_{12} = k_{LTA_{syn}} \cdot {}^{(Ca)}F_{HP}$$

$$r_5 = V_5 + V_{13} = \left(k_r + k_{r2} \cdot \frac{Ca}{K_{d2}^{Ca}}\right) \cdot \frac{{}^{(Ca)}F_2}{\Delta_2^{Ca}} - \left(\frac{k_r}{K_r} + \frac{k_{r2}}{K_{r2}} \cdot \frac{Ca}{K_{d3}^{Ca}}\right) \cdot \frac{{}^{(Ca)}F_{(Z)}}{\Delta_z^{Ca}}$$

$$= \rho_2 \frac{{}^{(Ca)}F_2}{\Delta_2^{Ca}} - \frac{\rho_2}{K_r} \frac{{}^{(Ca)}F_{(Z)}}{\Delta_z^{Ca}}$$

$$r_6 = V_6 + V_{14} = \left(k_{OX} + k_{OX2} \cdot \frac{Ca}{K_{d2}^{Ca}}\right) \cdot \frac{HPETE}{\Delta_2^{Ca}} \cdot {}^{(Ca)}F_2 - \left(\frac{k_{OX}}{K_{OX}} + \frac{k_{OX2}}{K_{OX2}} \cdot \frac{Ca}{K_{d3}^{Ca}}\right) \cdot \frac{HETE}{\Delta_Z^{Ca}} \cdot {}^{(Ca)}F_{(Z)}$$

$$= \rho_1 \frac{HPETE \cdot {}^{(Ca)}F_2}{\Delta_2^{Ca}} - \frac{\rho_1}{K_{OX}} \frac{HETE \cdot {}^{(Ca)}F_{(Z)}}{\Delta_z^{Ca}}$$

$$\acute{r_6} = \acute{V_6} + \acute{V_{14}} = \left(k_{OX} + k_{OX2} \cdot \frac{Ca}{K_{d2}^{Ca}}\right) \cdot \frac{LOOH}{\Delta_2^{Ca}} \cdot {}^{(Ca)}F_2 - \left(\frac{k_{OX}}{K_{OX}} + \frac{k_{OX2}}{K_{OX2}} \cdot \frac{Ca}{K_{d3}^{Ca}}\right) \cdot \frac{LOH}{\Delta_Z^{Ca}} \cdot$$

$$= \rho_1 \frac{LOOH \cdot {}^{(Ca)}F_2}{\Delta_2^{Ca}} - \frac{\rho_1}{K_{OX}} \frac{HETE \cdot {}^{(Ca)}F_{(Z)}}{\Delta_z^{Ca}}$$

$$r_7 = V_8 + V_{15} = k_z \cdot \frac{Z}{K_{dz}} \cdot \frac{\Delta_3^{Ca}}{\Delta_z^{Ca}} \cdot {}^{(Ca)}F_{(Z)} \qquad (1.12)$$

$$r_8 = V_{17} + V_{21} = k_{LTAin} \cdot LTA \cdot {}^{(Ca)}F_{AA}$$

$$r_9 = V_{18} + V_{22} + V_{20} + V_{24} = k_{LTAin} \cdot LTA \cdot {}^{(Ca)}F_{(Z)}$$

$$r_{10} = V_{19} + V_{23} = k_{LTAin} \cdot LTA \cdot {}^{(Ca)}F_{HP}$$

$$r_{11} = V_{25} + V_{26} = k_{LTAin} \cdot LTA \cdot {}^{(Ca)}F_2$$

Using these expressions for rates of slow reactions ODE system (1.3) can be presented in following manner:

$$e_1)\ \frac{d^{(Ca)}F_2}{dt} = r_7 - r_5 - r_6 - \acute{r_6} - r_{11}$$

$$e_3)\ \frac{d^{(Ca)}F_{(Z)}}{dt} = -r_1 + r_5 + r_6 + \acute{r_6} - r_4 + r_3 - r_7 - r_9$$

$$e_5)\ \frac{d\ {}^{(Ca)}F_{AA}}{dt} = r_1 - r_2 - r_8 \qquad (1.13)$$

$$e_7)\ \frac{d\ {}^{(Ca)}F_{HP}}{dt} = r_2 + r_4 - r_3 - r_{10}$$

System (1.13) describes dynamics of “partially reduced incomplete” catalytic cycle of 5LO which is presented in Fig A2 (part of the scheme selected with dashed green line).

Appendix 1.2. Description and reduction of “partially reduced” cycle.

At the next stage we supplement “partially reduced incomplete” catalytic cycle with all remaining states and transitions which have been omitted at the stage of construction of “incomplete” catalytic cycle. Taking into account all rules underlying modifications of rate equations describing processes of initial (non-reduced) catalytic cycle and resulted from reductions caused by fast Ca^{2+} and Z binding, “partially reduced” catalytic cycle of 5LO has been reconstructed on the basis of “partially reduced incomplete” catalytic cycle (Fig A2 in dashed line).

Fig. A2 represents the scheme of the “partially reduced” catalytic cycle of 5LO which includes (i) competitive binding and transformation of AA, HPETE and HETE at catalytic site, (ii) Fe2+

→ Fe3+ transition, (iii) competitive binding of non-redox inhibitor PF and (iv) inhibitory binding of AA at regulatory site. System of ordinary differential equations corresponding to the scheme presented below:

$$\frac{d^{(Ca)}F_2}{dt} = R_I - r_{11}$$
$$\frac{d^{(Ca)}F_{(Z)}^0}{dt} = -R_I - r_9 - r_1 - r_{17} - r_{13} - r_{20} - r_4 + r_3$$
$$\frac{d^{(Ca)}F_{AA}^0}{dt} = r_{10} - r_{16} - r_8 - r_2$$
$$\frac{d^{(Ca)}F_{HP}^0}{dt} = r_4 - r_{14} - r_{10} - r_3 + r_2$$
$$\frac{d^{(Ca)}F_{HT}^0}{dt} = r_{17} - r_{28} - r_{18}$$
$$\frac{d^{(Ca)}F_{PF}^0}{dt} = r_{20} - r_{29} - r_{21} \qquad (1.14)$$
$$\frac{d^{(Ca)}F_{AA}^{AA}}{dt} = r_{16} - r_{23} + r_{15}$$
$$\frac{d^{(Ca)}F_{3}^{AA}}{dt} = r_{13} - r_{15} - r_{19} - r_{27} - r_{22} - r_{12}$$
$$\frac{d^{(Ca)}F_{HP}^{AA}}{dt} = r_{12} - r_{24} + r_{14}$$
$$\frac{d^{(Ca)}F_{HT}^{AA}}{dt} = r_{18} - r_{25} + r_{19}$$
$$\frac{d^{(Ca)}F_{PF}^{AA}}{dt} = r_{22} - r_{26} + r_{21}$$
$$where\ R_I = r_7 - r_5 - r_6 - r_6'$$

Here, designations ${}^{X}F_{Y}^{U}$, X∈{(Ca)}, Y∈{2, 3, AA, HT, HP, PF, (Z)}, U∈{∅, A} are explained in Fig. 1 of main text and stand for states of 5LO. X takes only (Ca) value and describes that each state with the index is combination of two states with and without Ca^{2+} bound (see similar notations in eq. (1.5)). Y describes state of catalytic site of enzyme: “2” and “3” mean that Fe located in active site of the 5LO is either in reduced (Fe^{2+}) or oxidized (Fe^{3+}) state; AA, HP, HT, and PF mean that arachidonic acid, 5HPETE, 5HETE or PF are bound to catalytic site; (Z) means combination of two states with and without Z bound (see similar notations in eq. (1.5)). U describes state of regulatory site of enzyme: “∅” and “AA” mean that either nothing or

arachidonic acid bound to the regulatory site of the 5LO. r_i, i=1,…31, designate rate equations for transitions which can be written down similar to (1.12).

In accordance with available experimental facts and assumptions described above we can consider processes of AA binding to both catalytic and regulatory sites (r_i, i=1,13,14,15,16,18, 21), HT (r_i, i=17, 19) and non-redox inhibitor (PF) binding (r_i, i=20, 22) to catalytic site of 5LO as fast processes. This allows us to reduce systems of differential equations (1.14) assuming that k_i, i=1,13-22 are much larger than other rate constants. Indeed, summing up equations of the system (1.14) in such a way to exclude “fast” rates from right hand sides of as much numbers of differential equations as possible and tending k_i, i=1,13-22 to infinity we transform ODE system (1.14) to following reduced system of algebra differential equations:

$$e1)\ \frac{dF_2^{o\,Ca}}{dt} = R_I - r_{11}$$

$$e2)\ \frac{d\,{}^{(Ca)}F_{(L)}^{(AA)}}{dt} = -R_I - r_9 - r_4 + r_3 - r_8 - r_2 - r_{28} - r_{29} - r_{23} - r_{27} - r_{12} - r_{25} - r_{26}$$

$$= R_I - r_2 + r_3 - r_4 - r_8 - r_9 - r_{28} - r_{29} - r_{23} - r_{27} - r_{25} - r_{26} - r_{12} \qquad (1.15)$$

$$e3)\ r_1 = r_{14} = r_{17} = r_{20} = r_{16} = r_{18} = r_{13} = r_{21} = r_{15} = r_{19} = r_{22} = 0$$

$$e4)\ \frac{d\left({}^{(Ca)}F_{HP}^{(AA)}\right)}{dt} = r_4 - r_{10} = r_3 + r_2 + r_{12} - r_{24} = r_2 - r_3 + r_4 - r_{10} - r_{24} + r_{12}$$

Here, ${}^{(Ca)}F_{(L)}^{(AA)}$, ${}^{(Ca)}F_{HP}^{(AA)}$ are new variables of the ODE system designating sums of states of the enzyme which are interconnected by fast processes in catalytic cycle (see Figs A2):

$${}^{(Ca)}F_{(L)}^{(AA)} = {}^{(Ca)}F_{(Z)}^{0} + {}^{(Ca)}F_{AA}^{0} + {}^{(Ca)}F_{HT}^{0} + {}^{(Ca)}F_{PF}^{0} + {}^{(Ca)}F_{3}^{AA} + {}^{(Ca)}F_{AA}^{AA} + {}^{(Ca)}F_{HT}^{AA} + {}^{(Ca)}F_{PF}^{AA}$$

$${}^{(Ca)}F_{HP}^{(AA)} = {}^{(Ca)}F_{HP}^{0} + {}^{(Ca)}F_{HP}^{AA} \qquad (1.16)$$

Using equations (e3) of system (1.15) and algebraic equations (1.16) we can express all variables appearing in system (1.14) in terms of new variables (appearing in left hand side of differential equations of system (1.15)):

$${}^{(Ca)}F_{(Z)}^{0} = \frac{{}^{(Ca)}F_{(L)}^{(AA)}}{\mathfrak{d}_L}$$

$${}^{(Ca)}F_{(3)}^{AA} = \frac{AA}{K_{AA}^{i} \cdot \frac{\Delta_Z}{\Delta_3^{Ca}}} \cdot \frac{{}^{(Ca)}F_{(L)}^{(AA)}}{\mathfrak{d}_L}$$

$$
{}^{(Ca)}F_{AA}^{0} = \frac{AA}{K_{AA} \cdot \frac{\Delta_Z}{\Delta_3}} \cdot \frac{{}^{(Ca)}F_{(L)}^{(AA)}}{\eth_L}
$$

$$
{}^{(Ca)}F_{AA}^{AA} = \frac{AA}{K_{AA}} \cdot \frac{AA}{K_{AA}^{i} \cdot \frac{\Delta_Z}{\Delta_3^{Ca}}} \cdot \frac{{}^{(Ca)}F_{(L)}^{(AA)}}{\eth_L}
$$

$$
{}^{(Ca)}F_{HT}^{0} = \frac{HT}{K_{HT} \cdot \frac{\Delta_Z}{\Delta_3^{Ca}}} \cdot \frac{{}^{(Ca)}F_{(L)}^{(AA)}}{\eth_L}
$$

$$
{}^{(Ca)}F_{HT}^{AA} = \frac{HT}{K_{HT}} \cdot \frac{AA}{K_{AA}^{i} \cdot \frac{\Delta_Z}{\Delta_3^{Ca}}} \cdot \frac{{}^{(Ca)}F_{(L)}^{(AA)}}{\eth_L} \qquad (1.17)
$$

$$
{}^{(Ca)}F_{PF}^{0} = \frac{PF}{K_{PF} \cdot \frac{\Delta_Z}{\Delta_3^{Ca}}} \cdot \frac{{}^{(Ca)}F_{(L)}^{(AA)}}{\eth_L}
$$

$$
{}^{(Ca)}F_{PF}^{AA} = \frac{PF}{K_{PF}} \cdot \frac{AA}{K_{AA}^{i} \cdot \frac{\Delta_Z}{\Delta_3^{Ca}}} \cdot \frac{{}^{(Ca)}F_{(L)}^{(AA)}}{\eth_L}
$$

$$
{}^{(Ca)}F_{HP}^{0} = \frac{{}^{(Ca)}F_{HP}^{(AA)}}{\eth_{HP}}
$$

$$
{}^{(Ca)}F_{HP}^{AA} = \frac{AA}{K_{AA}^{i}} \cdot \frac{{}^{(Ca)}F_{HP}^{(AA)}}{\eth_{HP}}
$$

$$
\eth_L = 1 + \frac{AA}{K_{AA}^{i} \cdot \Delta_Z} + \eth_{HP} \cdot \left(\frac{AA}{K_{AA}} + \frac{HETE}{K_{HT}} + \frac{PF}{K_{PF}}\right) \cdot \frac{1}{\Delta_Z}
$$

$$
\eth_{HP} = 1 + \frac{AA}{K_{AA}^{i}}
$$

Slow reaction rates appearing in right hand sides of differential equations of system (1.15) can be grouped in following way:

$g_1=r_2$

$g_2=R_I$

$g_3=r_3+r_{12}$

$g_4=r_4$ (1.18)

$g_5=r_8+r_9+r_{28}+r_{29}+r_{23}+r_{27}+r_{25}+r_{26}$

$g_6=r_{10}+r_{24}$

$g_7=r_{11}$

Taking into account expressions (1.17) we can rewrite slow reaction rates listed in (1.18) in terms of new variables:

$$g_1 = k_{lo} * \frac{AA}{K_{AA} * \Delta_Z} * \frac{{}^{(Ca)}F_{(L)}^{(AA)}}{\eth_L}$$

$$g_2 = \left(\rho_2 + \rho_1 * (LOOH + HPETE)\right) * {}^{(Ca)}F_2 \; - \left(\frac{\rho_2}{K_r} + \frac{\rho_1}{K_{ox}} * (LOH + HETE) + \rho_7 * Z\right) \cdot \frac{F_{(L)}^{(A)(Ca)}}{\eth_L}$$

$$g_3 = k_3 * \left(\frac{HPETE}{K_{d3} * \Delta_Z} * \eth_{HP} * \frac{{}^{(Ca)}F_{(L)}^{(AA)}}{\eth_L} - {}^{(Ca)}F_{HP}^{(AA)}\right)$$

$$g_4 = k_{LTAsyn} * \frac{{}^{(Ca)}F_{HP}^{(AA)}}{\eth_{HP}} \qquad (1.19)$$

$$g_5 = k_{LTAin} * LTA \; * {}^{(Ca)}F_{(L)}^{(AA)}$$

$$g_6 = k_{LTAin} * LTA \; * {}^{(Ca)}F_{(HP)}^{(AA)}$$

$$g_7 = k_{LTAin} * LTA \; * {}^{(Ca)}F_2$$

Appendix 1.3. Derivation of the equations for the reduced catalytic cycle and rate equations.

Using these expressions for rates of slow reactions ODE system (1.15) can be presented in following manner:

$$e1)\; dF_2^{oCa}/dt = g_2 - g_7$$

$$e2)\; \frac{d\left({}^{(Ca)}F_{(L)}^{(AA)}\right)}{dt} = -g_2 - g_1 + g_4 - g_3 - \; g_5 \qquad (1.20)$$

$$e4)\; \frac{d\left({}^{(Ca)}F_{HP}^{(AA)}\right)}{dt} = -r_4 - r_{10} + r_3 + r_2 + r_{12} - r_{24} = g_1 - g_4 + g_3 - g_6$$

System (1.18) describes dynamics of “reduced” catalytic cycle of 5LO. Corresponding scheme of the “reduced” catalytic cycle is presented on Fig. 3 of the main text.

Thus, we have reduced catalytic cycle of 5LO from 26 states to 3 states. These 3 states are interconnected by 7 reactions: 4 reactions (g_{1-4}) describe transitions between states and 3 reactions (g_{5-7}) describe inactivation of the 5LO. Corresponding ODE system includes 3 variables and 18 parameters. To derive rate equations describing various activities of 5LO we assume that reactions g_{5-7} describing the enzyme inactivation are equal to zero. In this case the

ODE system has conservation law and one of the differential equation can be replaced with linear algebraic describing conservation of total active enzyme concentration. As a result we have come to new system of algebra – differential equations:

$$e1)\ d^{(Ca)}F_2\ /dt = g_2$$

$$e2)\ {}^{(Ca)}F_{(L)}^{(AA)} + {}^{(Ca)}F_{HP}^{(AA)} + {}^{(Ca)}F_2^{(0)} = F_a \qquad (1.21)$$

$$e3)\ d({}^{(Ca)}F_{HP}^{(AA)})/dt = g_1 - g_4 + g_3$$

Here, F_a stands for total concentration of active enzyme.

To derive rate equations characterizing different aspects of 5LO operation we can use quasi steady state approach. Indeed, to find expressions for steady state concentrations of enzyme states we assume left hand sides of equations (e1) and (e3) of system (1.19) to be equal to zero. As a result system of algebra-differential equations (1.21) transforms to system of algebraic equations which solution can be written in following manner:

$$\frac{{}^{(Ca)}F_{(L)}^{(AA)}}{\mathfrak{d}_L} = \frac{F_a}{\mathfrak{d}_L + \Delta_{HP} + \Delta_{redox}} = \frac{F_a}{\Delta_{tot}} \qquad (1.22)$$

$${}^{(Ca)}F_2 = \Delta_{redox} \cdot \frac{{}^{(Ca)}F_{(L)}^{(AA)}}{\mathfrak{d}_L}—, \qquad (1.23)$$ где

$${}^{(Ca)}F_{HP}^{(AA)} = \frac{{}^{(Ca)}F_{(L)}^{(AA)}}{\mathfrak{d}_L} \cdot \Delta_{HP} \qquad (1.24)$$

where

$$\Delta_{HP} = \frac{\mathrm{k_{lo}} \cdot \frac{\mathrm{AA}}{K_{AA}} + \frac{k_3}{K_{d3}} \cdot HP \cdot \mathfrak{d}_{HP}}{\Delta_Z \cdot \left(\frac{k_{LTAsyn}}{\mathfrak{d}_{HP}} + k_3\right)} \qquad (1.25)$$

$$\Delta_{redox} = \frac{\frac{\rho_2}{K_r} + \frac{\rho_1}{K_{ox}} * (LOH - HT) + \rho_7 * Z}{\left(\rho_2 + \rho_1 * (LOOH + HP)\right)} \frac{\Delta_Z^{Ca}}{\Delta_2^{Ca}} \qquad (1.26)$$

Substitution of these expressions to corresponding rate equations results in following expressions for dependence of key activities of 5LO on its substrates, products and inhibitors concentrations:

Consumption of arachidonic acid in oxygenase reaction:

$$V_{AAcons}^{5LO} = g_1 = \mathrm{k_{lo}} * \frac{\mathrm{AA}}{K_{AA} * \Delta_Z} \frac{F_a}{\Delta_{tot}} \qquad (1.27)$$

LTA synthesis:

$$V_{LTAsyn}^{5LO} = g_2 = k_{LTAsyn} * \frac{{}^{(Ca)}F_{HP}^{(AA)}}{\mathfrak{d}_{HP}} = k_{LTAsyn} \cdot \Delta_{HP} \frac{F_a}{\Delta_{tot} \cdot \mathfrak{d}_{HP}} \qquad (1.28)$$

HP production in oxigenase reaction:

$$V^{5LO}_{HPETEsyn} = -g_3 = -k_3 \cdot \frac{F_a}{\Delta_{tot}} \cdot \left(\frac{HP}{\Delta_Z \cdot K_{d3}} \cdot \eth_{HP} - \Delta_{HP}\right) \qquad (1.29)$$

HP consumption (HT production) in pseudoperoxidase reaction:

$$V^{5LO}_{HPETEcons} = \rho_1 * HP * F_2^{(Ca)} - \frac{\rho_1}{K_{ox}} * HT * \frac{{}^{(Ca)}F^{(AA)}_{(L)}}{\eth_L} = \frac{F_a}{\Delta_{tot}} \cdot \rho_1 \cdot \left(HP \cdot \Delta_{redox} - \frac{HT}{K_{ox}}\right) \quad (1.30)$$

$$V^{5LO}_{HETEsyn} = V^{5LO}_{HPETEcons} \qquad (1.31)$$

HP consumption (HT production) in pseudoperoxidase reaction:

$$V^{5LO}_{LOOHcons} = \rho_1 \cdot LOOH \cdot F_2^{(Ca)} - \frac{\rho_1}{K_{ox}} \cdot LOH \cdot \frac{{}^{(Ca)}F^{(AA)}_{(L)}}{\eth_L} =$$

$$= \frac{F_a}{\Delta_{tot}} \cdot \rho_1 \cdot \left(LOOH \cdot \Delta_{redox} - \frac{LOH}{K_{ox}}\right) \qquad (1.32)$$

$$V^{5LO}_{LOHsyn} = V^{5LO}_{LOOHcons} \qquad (1.33)$$

Here, following designations have been used:

$$\Delta_Z = 1 + \frac{Z}{K_{dz}}, \quad \Delta_2^{Ca} = 1 + \frac{Ca}{K_{dz}^{Ca}}, \quad \Delta_3^{Ca} = 1 + \frac{Ca}{K_{d3}^{Ca}}, \quad \Delta_Z^{Ca} = \Delta_Z * \Delta_3^{Ca},$$

$$\eth_L = 1 + \frac{AA}{K_{AA}^{i} * \Delta_Z} + \eth_{HP} * \left(\frac{AA}{K_{AA}} + \frac{HETE}{K_{HT}} + \frac{PF}{K_{PF}}\right) * \frac{1}{\Delta_Z}$$

$$\eth_{HP} = 1 + \frac{AA}{K_{AA}^{i}},$$

Appendix 1.4 Additional assumptions on relationships between parameters of the model

$$K_{AA}^{iAA} = K_{AA}^{i}$$

$$K_{HT}^{iAA} = K_{HT} \qquad (1.33)$$

$$K_{HP}^{iAA} = K_{AA}^{i}$$

Appendix 1.5 ODE system of leukotriene biosynthesis pathway

$$\frac{dAA}{dt} = -V^{5LO}_{AAcons} \qquad (1.34)$$

$$\frac{dLTA4}{dt} = V^{5LO}_{LTAsyn} \qquad (1.35)$$

$$\frac{d5HP}{dt} = V^{5LO}_{HPsyn} - V^{5LO}_{HPcons} \qquad (1.36)$$

$$\frac{dHT}{dt} = V^{5LO}_{HPcons} \qquad (1.37)$$

$$\frac{dLOOH}{dt} = -V_{LOOHcons}^{5LO} \quad (1.38)$$

$$\frac{dF^a}{dt} = -V_{lta\_in}^{5LO} \quad (1.39)$$

$$LOOH + LOH = LOOH_0 \quad (1.40)$$

$$F^a + F^i = E_0, \quad (1.41)$$

Appendix 2. Kinetic model of phospholipase A_2.

On the basis of several models of the enzyme developed previously and available experimental data [29, 30] we have derived the equation describing the rate of PLA2:

$$V_{AA}^{PLA2} = \frac{V_{\max}^{PLA_2} \cdot PLA_2^{Ca} \cdot PL}{K_m^{PLA_2\_APC} + PL}, \quad (2.1)$$

where $PLA_2^{Ca} = \frac{Ca}{K_{Ca}^{PLA2} + Ca}$

Appendix 3. Kinetic model of Glutathione peroxidase

ODE system describing catalytic cycle of gluthation peroxidase (Fig A3) can be represented in following way:

$$\frac{d[GPX]}{dt} = V_5 - V_6$$

$$\frac{d[GPX_{HP}]}{dt} = V_6 - V_1$$

$$\frac{d[GPX_{GSSG}]}{dt} = V_2 - V_5 \quad (3.1)$$

$$\frac{d[GPX']}{dt} = V_3 - V_4$$

$$\frac{d[GPX'_{HT}]}{dt} = V_1 - V_3$$

$$\frac{d[GPX'_{2*GSH}]}{dt} = V_4 - V_2$$

All reaction rates are in accordance to mass action law. Let us assume that reactions of substrate binding and product dissociation (Vi, i=3,4,5,6) are much faster than rates of catalytic steps (V1 and V2):

$$V_1 = k_1^{gpx} \cdot [GPX_{HP}]$$

$$V_2 = k_2^{gpx} \cdot [GPX'_{2*GSH}]$$

$$V_3 = k_3 \cdot w_3 = k_3 \cdot ([GPX'_{HT}] - \frac{[GPX'] \cdot [HETE]}{K_{HT}^{gpx}})$$

$$V_4 = k_4 \cdot w_4 = k_4 \cdot (\frac{[GPX'] \cdot [GSH] \cdot [GSH]}{K_{GSH}^{gpx} \cdot K_{GSH}^{gpx}} - [GPX'_{2*GSH}]) \quad (3.2)$$

$$V_5 = k_5 \cdot w_5 = k_5 \cdot ([GPX_{GSSG}] - \frac{[GPX] \cdot [GSSG]}{K_{GSSG}^{gpx}})$$

$$V_6 = k_6 \cdot w_6 = k_6 \cdot (\frac{[GPX] \cdot [HP]}{K_{HP}^{gpx}} - [GPX_{HP}])$$

Summing up first 3 (and last 3) equations of system (3.1) and replacing first (fourth) equation with corresponding resulting equation, one obtains:

$$\frac{d([GPX] + [GPX_{HP}] + [GPX_{GSSG}])}{dt} = V_2 - V_1$$

$$\frac{d[GPX_{HP}]}{dt} = V_6 - V_1$$

$$\frac{d[GPX_{GSSG}]}{dt} = V_2 - V_5$$

$$\frac{d([GPX'] + [GPX'_{HT}] + [GPX'_{2*GSH}])}{dt} = V_1 - V_2 \qquad (3.3)$$

$$\frac{d[GPX'_{HT}]}{dt} = V_1 - V_3$$

$$\frac{d[GPX'_{2*GSH}]}{dt} = V_4 - V_2$$

Let us introduce new variables *X* and *Y*:

$$X = [GPX] + [GPX_{HP}] + [GPX_{GSSG}]$$

$$Y = [GPX'] + [GPX'_{HT}] + [GPX'_{2*GSH}] \qquad (3.4)$$

Tending k_i, i = 3, 4, 5, 6 to infinity and taking into account expressions for new variables, one obtains from system (3.3):

$$\frac{dX}{dt} = V_2 - V_1$$

$$\frac{dY}{dt} = V_1 - V_2 \qquad (3.5)$$

$$w_3 = 0, w_4 = 0, w_5 = 0, w_6 = 0$$

Old variables appearing in left hand side of system (3.1) can be expressed in terms of new variables (X and Y) on the basis of equations (3.4) and algebraic equations of system (3.5). As a result, one obtains following expressions:

$$[GPX_{HP}] = \frac{X}{\Delta_1} \cdot \frac{[HP]}{K_{HP}^{gpx}}$$

$$[GPX'_{HT}] = \frac{Y}{\Delta_2} \cdot \frac{[HT]}{K_{HT}^{gpx}}$$

$$[GPX'_{2*GSH}] = \frac{Y}{\Delta_2} \cdot \frac{[GSH] \cdot [GSH]}{K_{GSH}^{gpx} \cdot K_{GSH}^{gpx}} \qquad (3.7)$$

$$[GPX_{GSSG}] = \frac{X}{\Delta_1} \cdot \frac{[GSSG]}{K_{GSSG}^{gpx}},$$

где $\Delta_1 = 1 + \frac{[HP]}{K_{HP}^{gpx}} + \frac{[GSSG]}{K_{GSSG}^{gpx}}, \Delta_2 = 1 + \frac{[HT]}{K_{HT}^{gpx}} + \frac{[GSH] \cdot [GSH]}{K_{GSH}^{gpx} \cdot K_{GSH}^{gpx}}$

Imposing ODE system (3.5) under steady state conditions, one obtains:

$$V_1 = V_2 = V^{gpx}$$
$$X + Y = GPX_{full} \quad , \qquad (3.6)$$

where GPX_{full} is total concentration of gluthation peroxidase.

Substituting (3.7) to (3.6) and solving resulting system of linear algebraic equations, one derives enzyme reaction rate ($V^{gpx} = V_1 = V_2$) as a function of substrates (HP, GSH) and products (HETE, GSSG) concentrations, total enzyme concentration (GPX_{full}), dissociation ($K_{HP}^{gpx}, K_{HT}^{gpx}, K_{GSH}^{gpx}, K_{GSSG}^{gpx}$) and catalytic ($k_1^{gpx}, k_2^{gpx}$) constants:

$$V^{gpx} = \frac{GPX_{full} \cdot B}{A} \ ,$$

where

$$A = \frac{k_1^{gpx} \cdot HP}{K_{HP}^{gpx}} \cdot (1 + \frac{GSH \cdot GSH}{K_{GSH}^{gpx} \cdot K_{GSH}^{gpx}} + \frac{HT}{K_{HT}^{gpx}}) + \frac{k_2^{gpx} \cdot GSH \cdot GSH}{K_{GSH}^{gpx} \cdot K_{GSH}^{gpx}} \cdot (1 + \frac{HP}{K_{HP}^{gpx}} + \frac{GSSG}{K_{GSSG}^{gpx}})$$

$$B = \frac{k_1^{gpx} \cdot k_2^{gpx} \cdot HP \cdot GSH \cdot GSH}{K_{HP}^{gpx} \cdot K_{GSH}^{gpx} \cdot K_{GSH}^{gpx}} \qquad (3.8)$$

Applying techniques described elsewhere [28] we can express dissociation and catalytic constants in terms of turnover rates and Michaelis constants in following manner:

$$A = \frac{HP}{K_m^{HPETE}} \cdot (1 + \frac{HT}{K_{HT}^{gpx}}) + \frac{GSH \cdot GSH}{K_m^{GSH} \cdot K_m^{GSH}} \cdot (1 + \frac{GSSG}{K_{GSSG}^{gpx}}) + \frac{GSH \cdot GSH \cdot HP}{K_m^{GSH} \cdot K_m^{GSH} \cdot K_m^{HP}}$$

$$B = \frac{k_{cat}^{gpx} \cdot HP \cdot GSH \cdot GSH}{K_m^{HP} \cdot K_m^{GSH} \cdot K_m^{GSH}}$$

Thus, reaction rate of gluthation peroxidase includes 5 unknown parameters. Value of Michaelis constant with respect to gluthation (K_m^{GSH}) has been taken from literature. Other 4 parameters have been estimated on the basis of fitting of the rate equation agains two sets of experimental data. Figs 9 and 10 demonstrate quality of the fitting. Values of the parameters are summarized in Table 1.

Appendix 4. Kinetic model of the 5-hydroxyeicosanoid dehydrogenase

ODE system describing catalytic cycle of 5-hydroxyeicosanoid dehydrogenase (Fig A4) can be represented in following way:

$$\frac{d[HEDH]}{dt} = V_5 - V_6$$
$$\frac{d[HEDH_{HETE}]}{dt} = V_6 - V_1$$
$$\frac{d[HEDH_{NADPH}]}{dt} = V_2 - V_5 \qquad (4.1)$$
$$\frac{d[HEDH']}{dt} = V_3 - V_4$$

$$\frac{d[HEDH'_{oxoETE}]}{dt} = V_1 - V_3$$

$$\frac{d[HEDH'_{NADP}]}{dt} = V_4 - V_2$$

All reaction rates are in accordance to mass action law. Let us assume that reactions of substrate binding and product dissociation (Vi, i=3,4,5,6) are much faster than rates of catalytic steps (V1 and V2):

$$V_1 = k_1^{hedh} \cdot [HEDH_{HETE}] - k_{-1}^{hedh} \cdot [HEDH'_{oxoETE}]$$

$$V_2 = k_2^{hedh} \cdot [HEDH'_{NADP}] - k_{-2}^{hedh} \cdot [HEDH_{NADPH}]$$

$$V_3 = k_3 \cdot w_3 = k_3 \cdot ([HEDH'_{oxoETE}] - \frac{[HEDH'] \cdot [oxoETE]}{K_{oxoETE}^{hedh}})$$

$$V_4 = k_4 \cdot w_4 = k_4 \cdot (\frac{[HEDH'] \cdot [NADP]}{K_{NADP}^{hedh}} - [HEDH'_{NADP}]) \qquad (4.2)$$

$$V_5 = k_5 \cdot w_5 = k_5 \cdot ([HEDH_{NADPH}] - \frac{[HEDH] \cdot [NADPH]}{K_{NADPH}^{hedh}})$$

$$V_6 = k_6 \cdot w_6 = k_6 \cdot (\frac{[HEDH] \cdot [HT]}{K_{HT}^{hedh}} - [HEDH_{HT}])$$

Summing up first 3 (and last 3) equations of system (4.1) and replacing first (fourth) equation with corresponding resulting equation, one obtains:

$$\frac{d([HEDH] + [HEDH_{HT}] + [HEDH_{NADPH}])}{dt} = V_2 - V_1$$

$$\frac{d[HEDH_{HT}]}{dt} = V_6 - V_1$$

$$\frac{d[HEDH_{NADPH}]}{dt} = V_2 - V_5$$

$$\frac{d([HEDH'] + [HEDH'_{oxoETE}] + [HEDH'_{NADP}])}{dt} = V_1 - V_2 \qquad (4.3)$$

$$\frac{d[HEDH'_{oxoETE}]}{dt} = V_1 - V_3$$

$$\frac{d[HEDH'_{NADP}]}{dt} = V_4 - V_2$$

Let us introduce new variables *X* and *Y*:

$$X = [HEDH] + [HEDH_{HT}] + [HEDH_{NADPH}]$$

$$Y = [HEDH'] + [HEDH'_{oxoETE}] + [HEDH'_{NADP}] \qquad (4.4)$$

Tending k_i, i = 3, 4, 5, 6 to infinity and taking into account expressions for new variables, one obtains from system (4.3):

$$\frac{dX}{dt} = V_2 - V_1$$

$$\frac{dY}{dt} = V_1 - V_2 \qquad (4.5)$$

$$w_3 = 0, w_4 = 0, w_5 = 0, w_6 = 0,$$

Applying to the system (4.5) the same techniques as we have applied to the system (3.5) of Appendix 3, one obtains following expression for rate equation of 5-hydroxyeicosanoid dehydrogenase:

$$V^{hedh} = \frac{HEDH_{full} \cdot B}{A} \qquad (4.8),$$

where

$$B = \frac{k_1^{hedh} \cdot k_2^{hedh} \cdot HT \cdot NADP}{K_{HT}^{hedh} \cdot K_{NADP}^{hedh}} - \frac{k_{-1}^{hedh} \cdot k_{-2}^{hedh} \cdot oxoETE \cdot NADPH}{K_{oxoETE}^{hedh} \cdot K_{NADPH}^{hedh}}$$

$$A = ((1 + \frac{NADP}{K_{NADP}^{hedh}} + \frac{oxoETE}{K_{oxoETE}^{hedh}}) \cdot (\frac{k_1^{hedh} \cdot HT}{K_{HT}^{hedh}} + \frac{k_{-2}^{hedh} \cdot NADPH}{K_{NADPH}^{hedh}}) + \\ + (1 + \frac{HT}{K_{HT}^{hedh}} + \frac{NADPH}{K_{NADPH}^{hedh}}) \cdot (\frac{k_{-1}^{hedh} \cdot oxoETE}{K_{oxoETE}^{hedh}} + \frac{k_2^{hedh} \cdot NADP}{K_{NADP}^{hedh}}))$$

Thus, reaction rate of the enzyme includes 8 unknown parameters. These parameters have been estimated on the basis of fitting of the rate equation against two sets of experimental data. Figs 11 and 12 demonstrate quality of the fitting. Values of the parameters are summarized in Table 1.

Appendix 5. Metabolite degradation rates.

$$V_{HTd} = k_{HTd} \cdot HT$$

$$V_{LTAd} = k_{LTAd} \cdot LTA4 \qquad (5.1)$$

$$V_{oxoETEd} = k_{oxoETEd} \cdot oxoETE$$

Appendix 6.

Fig. A5 demonstrates comparison of model simulation results with experimental data [23] characterizing 5LO inactivation in the presence of LTA4. Despite model simulations poorly reproduces experimental data it is possible to conclude that deviations of model predictions from experimental points is less than two times. In spite of production of HP, HT and LTA4 [38] has not been perfectly described (Fig. A6), model satisfactory reproduces distribution of lipid peroxide products [38] 10 mins after addition of the enzyme to reaction media (Fig. A7). Time course of experimentally measured LTA4 production [26] has also been reproduced satisfactory by our model (Fig. A8).
