# Supplementary material for: Regulation of leukotriene and 5oxoETE synthesis and the effect of 5-lipoxygenase inhibitors: a mathematical modeling approach
Source: BMC Syst Biol. 2012 Nov 12;6:141. doi: 10.1186/1752-0509-6-141 (PMC3546923; doi:10.1186/1752-0509-6-141)
Supplement: Additional file 2 — Appendix 2. Additional figures. [file 1752-0509-6-141-S2.pdf]

Appendix figure legends

Figure A1. Scheme of the «incomplete» catalytic cycle. Designations: pink -the enzyme states (according to designations on Fig.1 of main text), blue – metabolites-variables of the model, yellow – species with buffered concentrations, dashed lines – reactions considered as fast on the first stage.

Figure A2. Complete catalytic cycle. Partially reduced incomplete cycle is shown in green dashed line. Designations: dashed lines – fast reactions, blue - metabolites-variables of the model, yellow – species with buffered concentrations. On the bottom part the processes of 5LO reduction-oxidation are shown (I).

Figure A3. The catalytic cycle of the glutathione-peroxidase (mechanism Ping-pong Bi Bi) GPX – glutathione-peroxidase, HPETE – 5-HPETE, HETE – 5-HETE, GSH –glutathione, GSSG –glutathione oxidized, GPX' – modified glutathione-peroxidase, GPX_X and GPX'_X (where X – HPETE, GSSG, 2*GSH, HETE) – complexes of enzyme with substrates and products. Dashed lines designate fast reaction, solid lines – slow reactions.

Figure A4. Catalytic cycle of 5-hydroxyeicosanoid dehydrogenase (mechanism Ping-Pong Bi-Bi). HEDH – 5-hydroxyeicosanoid dehydrogenase, HETE – 5-HETE, oxoETE – 5-oxoETE, HEDH' –modified 5-hydroxyeicosanoid dehydrogenase. Dashed lines designate fast reaction, solid lines – slow reactions.

Figure A5. Time dependent inactivation of 5LOX in presence of LTA4 from reference [23].

Figure A6. Formation of 5LO products by wt-5LO in the presence of CLP and PC [38]. Purified recombinant wt-5LO was incubated for 10 min at room temperature in the presence of AA (100 μM), Ca2+ (100 μM), ATP (1 mM), and 13-HPODE (10 μM).

Figure A7. 5LO and CLP reduce lipid hydroperoxides [38]. 5LO and_or CLP (stoichiometry 1:1) were incubated with 5-HPETE (25 μM) and 13-HPODE (10 μM), according to the HPLC assay protocol. Ca^{2+} (0.1 mM) and PC (25 μg/ml) were present in all incubations, whereas AA was omitted.

Figure A8. Time dependence of LTA4 concentration measured under following conditions: 10 μM of 5-HPETE, 1.5 μg/mL 5-Lox [26]. Dots correspond to experimental data, solid line is model generated curve.

Figure A9. Dependence of the rate of glutathione-peroxidase on 5-HPETE concentration [1A]. Squares – experimental data, solid line – modeling results.

Figure A10. Dependence of the 5-HETE concentration on time at the next initial conditions: 10 мкМ 5-HPETE, 5 мМ of reduced glutathione [ref. 1A]. Squares – experimental data, solid line – modeling results.

Figure A11. Dependence of the rate of 5-hydroxyeicosanoid dehydrogenase on 5-HETE concentration at different NADP concentrations (squares – experimental data, solid line – modeling results): 0.1 uM (black curve), 0.17 uM (blue curve), 0.3 uM (red curve), 0.6 uM (green curve), 1.5 uM (pink curve) and 10 uM (brown curve) [ref. 2A].

Figure A12. Dependence of the backward rate of the 5-hydroxyeicosanoid dehydrogenase, i.e. $V_{rev} = -V^{hedh}$, on 5-oxoETE concentration [ref. 2A].

References

1A. Jakobsson PJ, Mancini JA, Riendeau D, Ford-Hutchinson AW: **Identification and characterization of a novel microsomal enzyme with glutathione-dependent transferase and peroxidase activities.** *J Biol Chem.* 1997, **272**(36):22934-9.

2A. Erlemann KR, Cossette C, Grant GE, Lee GJ, Patel P, Rokach J, Powell WS.: **Regulation of 5-hydroxyeicosanoid dehydrogenase activity in monocytic cells.** *Biochem J.* 2007, **403**(1):157-65.

Figure A1

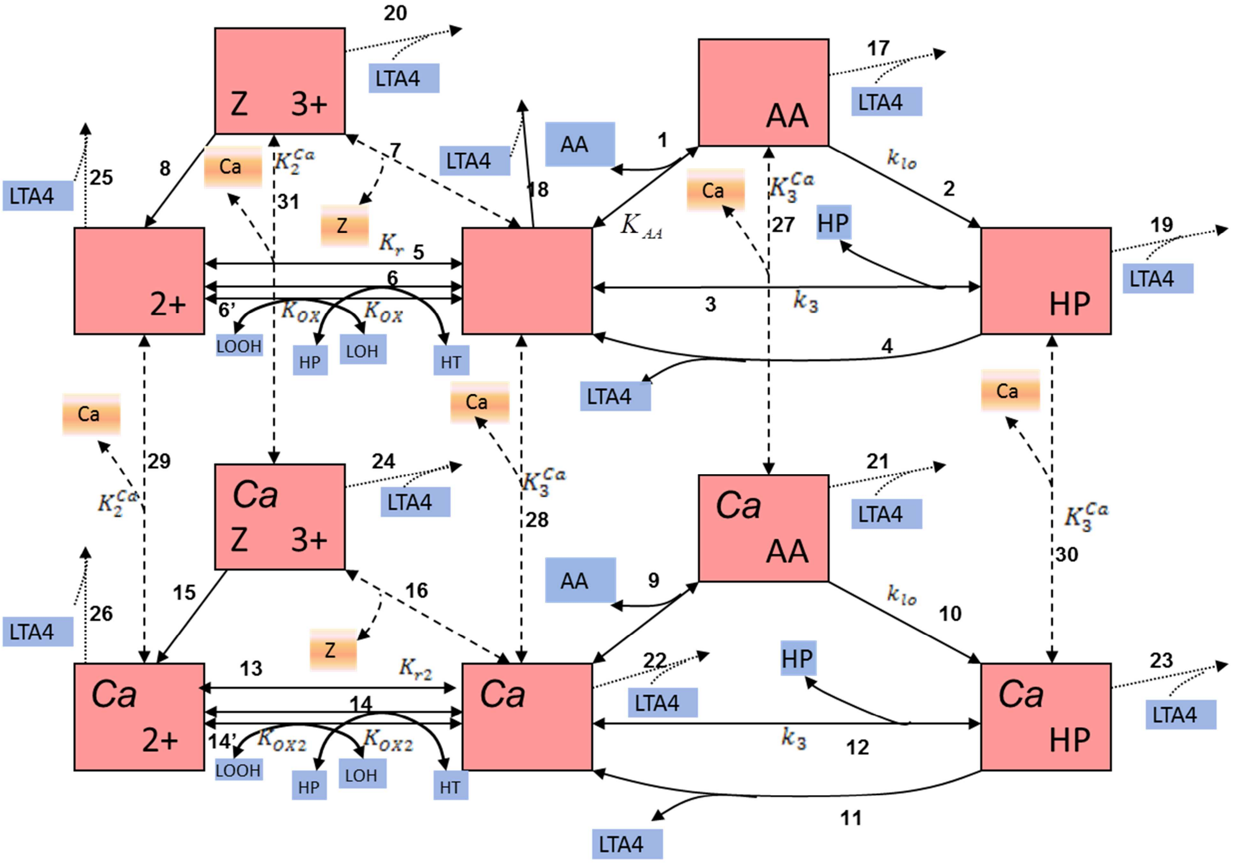

Z 3+
2+
AA
HP
Ca Z 3+
Ca 2+
Ca
Ca AA
Ca HP
LTA4
AA
HP
Ca
Z
LOOH
LOH
HT
K_{AA}
k_{lo}
k_3
K_r
K_{r2}
K_{OX}
K_{OX2}
K_2^{Ca}
K_3^{Ca}
1
2
3
4
5
6
6'
7
8
9
10
11
12
13
14
14'
15
16
17
18
19
20
21
22
23
24
25
26
27
28
29
30
31

Figure A2

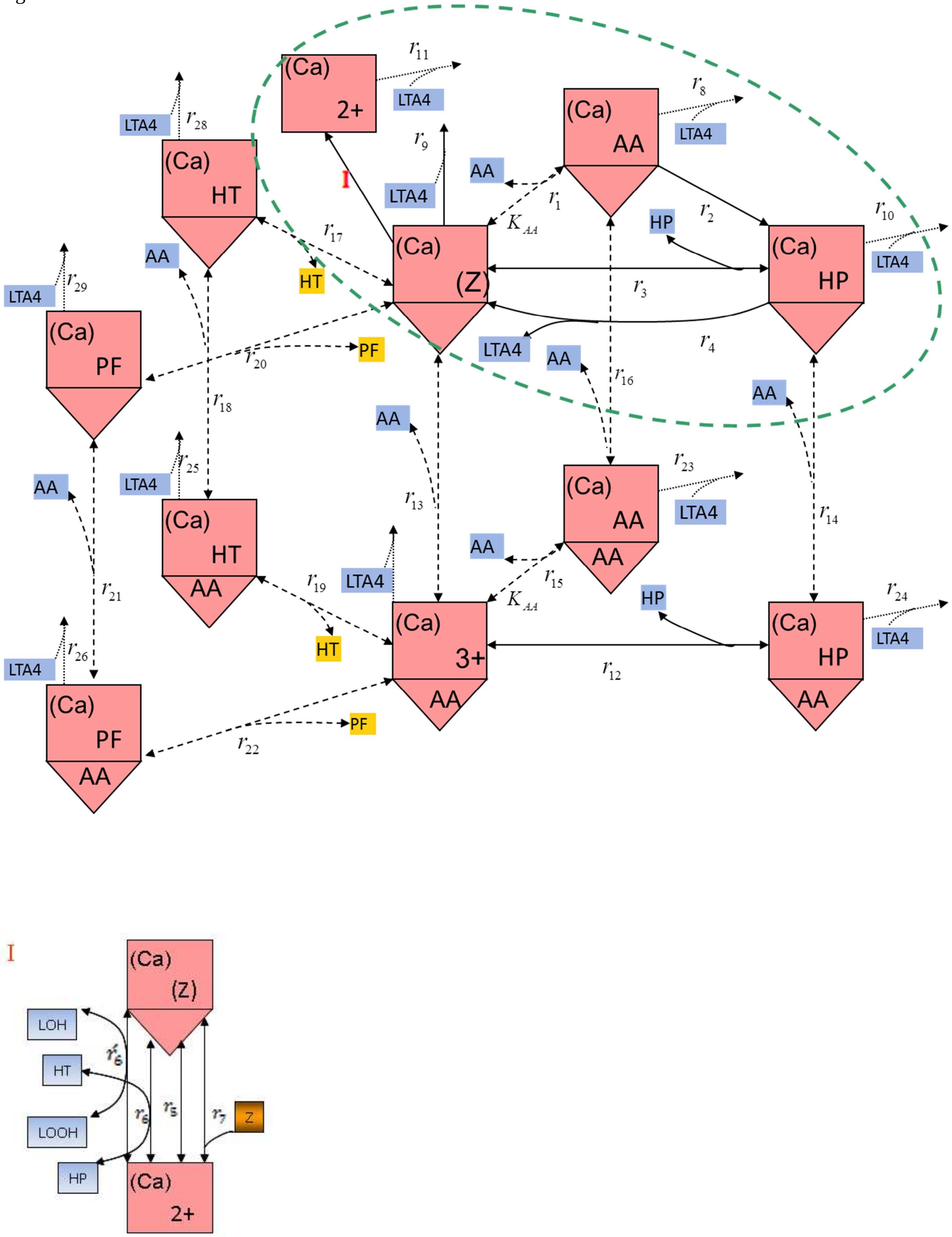

(Ca) 2+
(Ca) AA
(Ca) (Z)
(Ca) HP
(Ca) HT
(Ca) PF
(Ca) AA AA
(Ca) 3+ AA
(Ca) HP AA
(Ca) HT AA
(Ca) PF AA
LTA4
AA
HT
PF
HP
I
r_1
r_2
r_3
r_4
r_8
r_9
r_{10}
r_{11}
r_{12}
r_{13}
r_{14}
r_{15}
r_{16}
r_{17}
r_{18}
r_{19}
r_{20}
r_{21}
r_{22}
r_{23}
r_{24}
r_{25}
r_{26}
r_{28}
r_{29}
K_{AA}
(Ca) (Z)
(Ca) 2+
LOH
HT
LOOH
HP
Z
r_5
r_6
r_6'
r_7

Figure A3

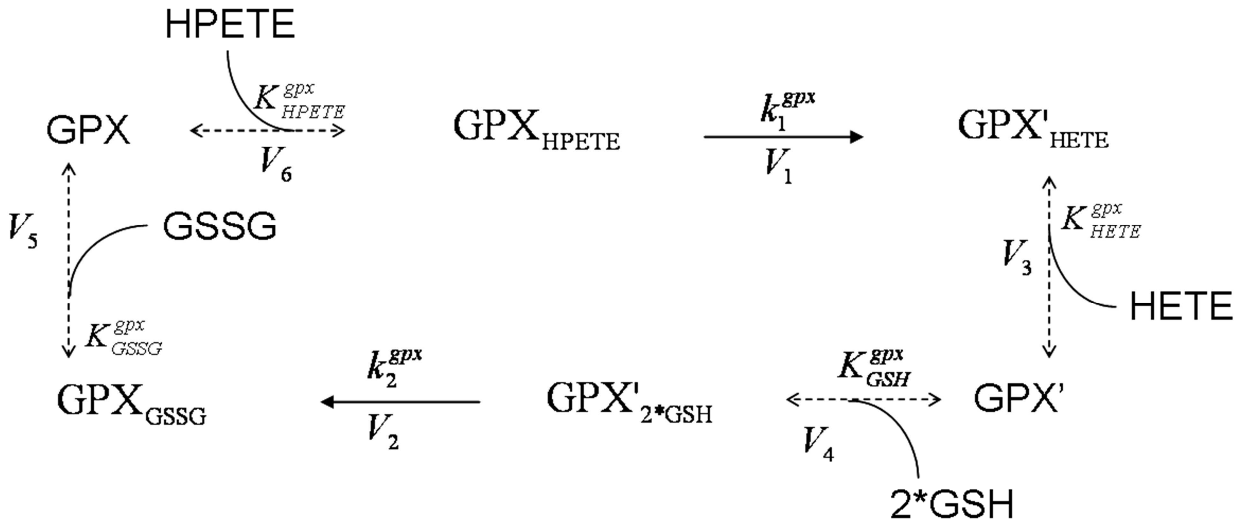

Figure A4

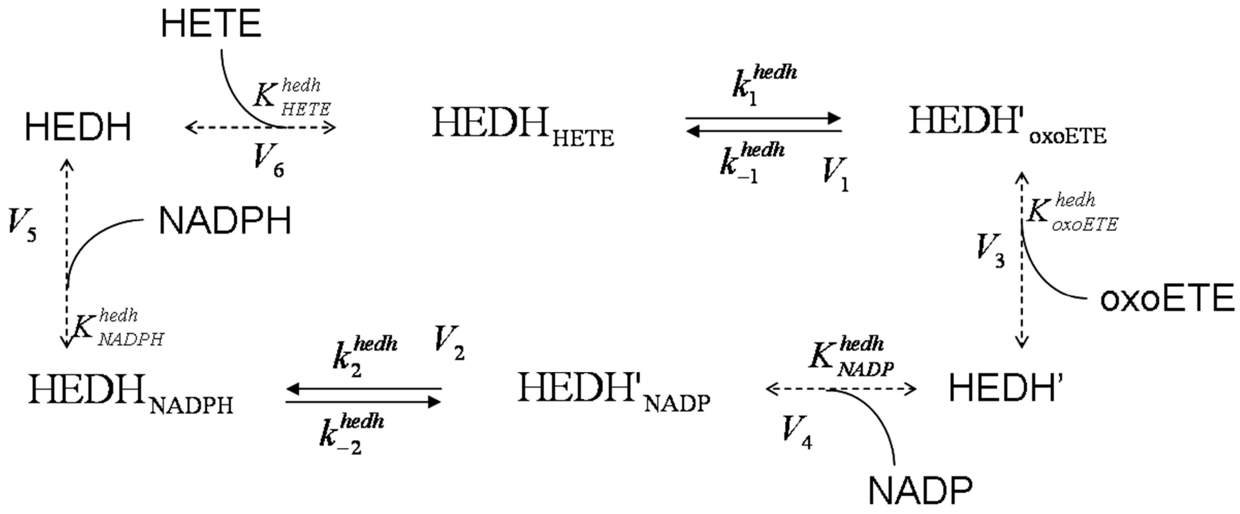

Figure A5

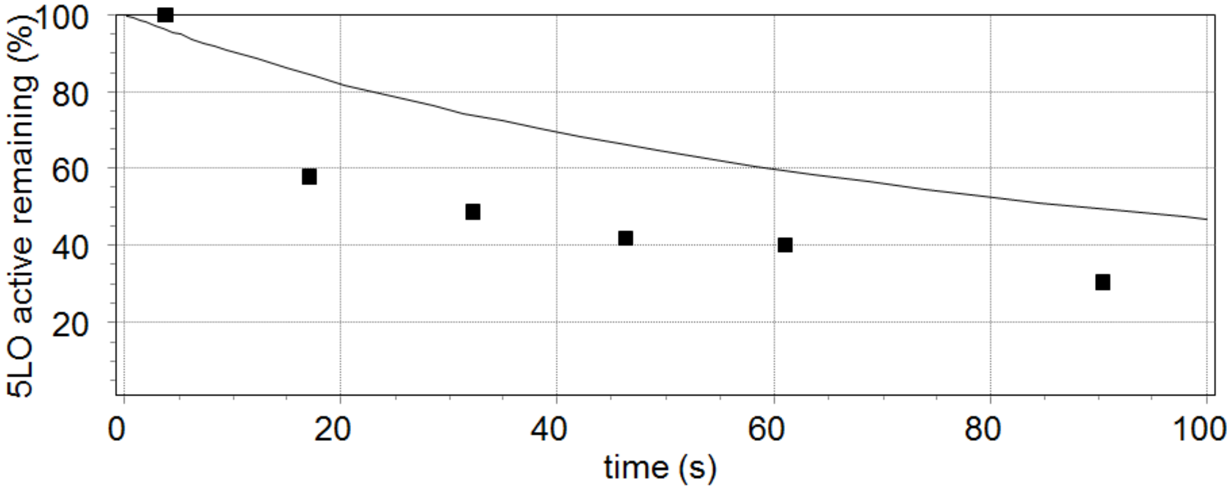

Figure A6

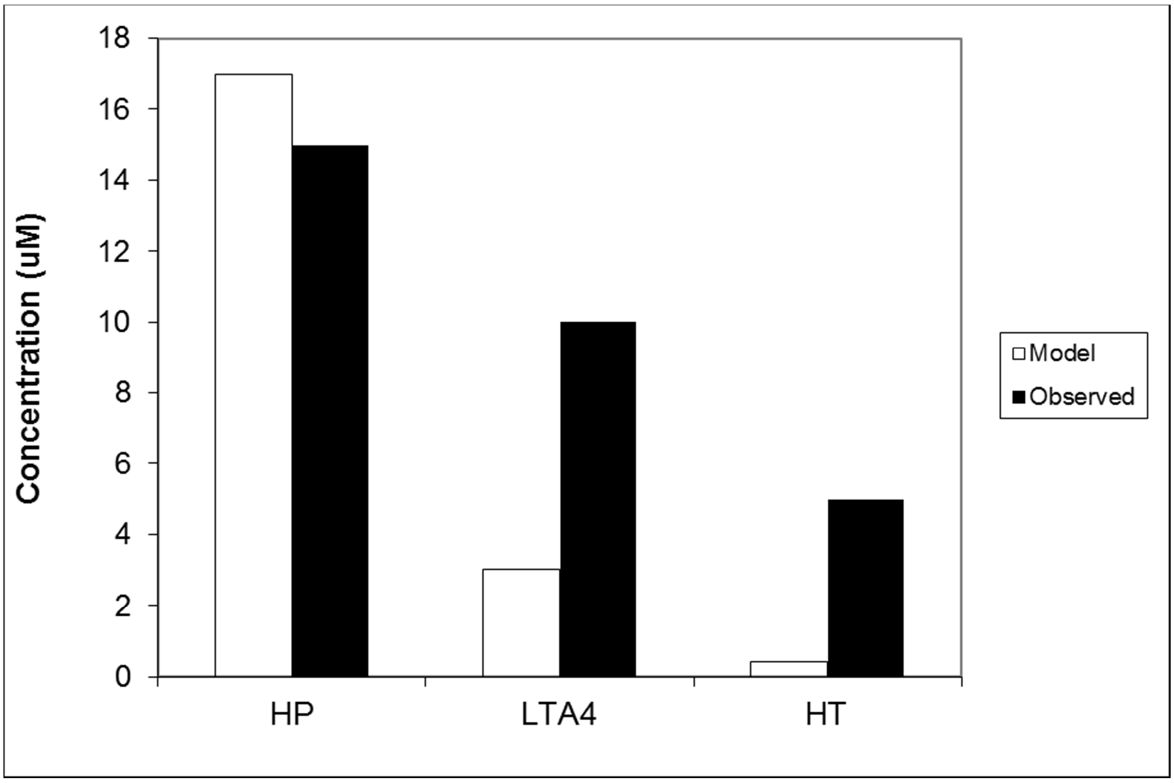

Figure A7.

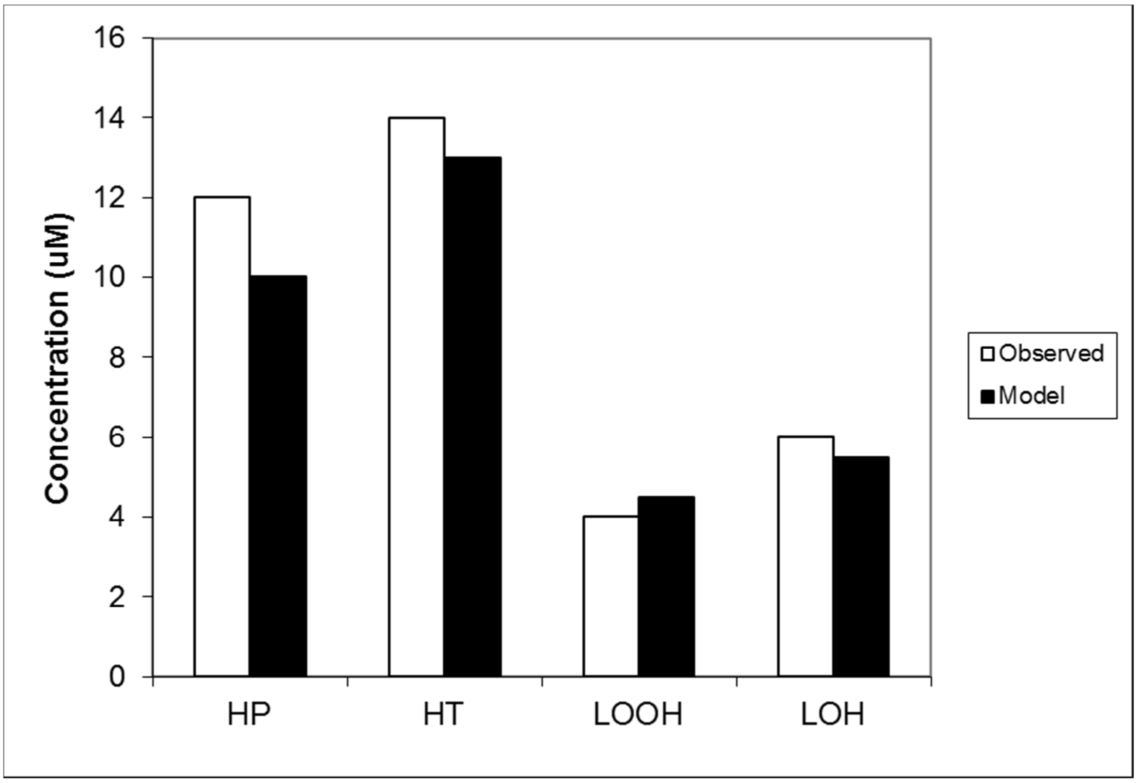

16
14
12
10
8
6
4
2
0
Concentration (uM)
HP
HT
LOOH
LOH
Observed
Model

Figure A8.

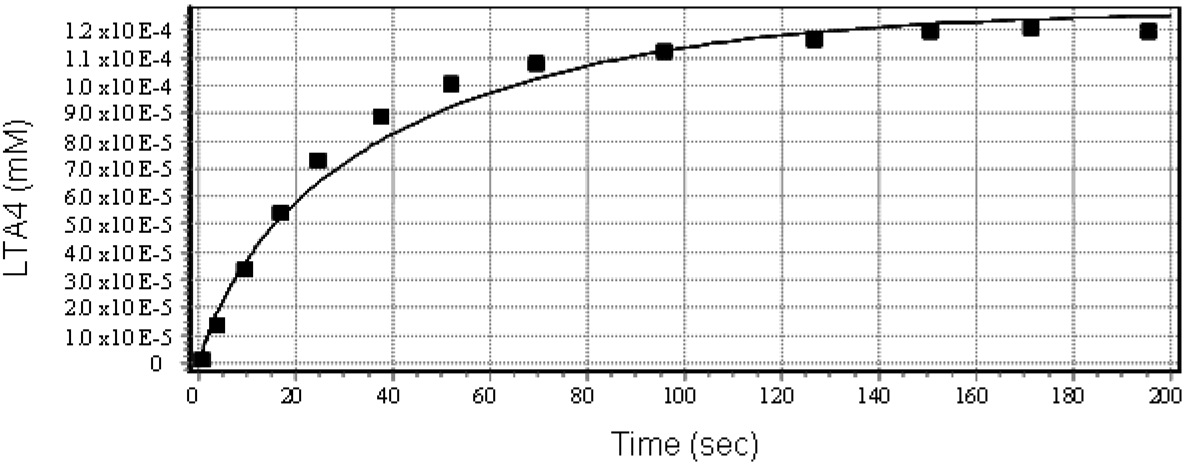

Figure A9

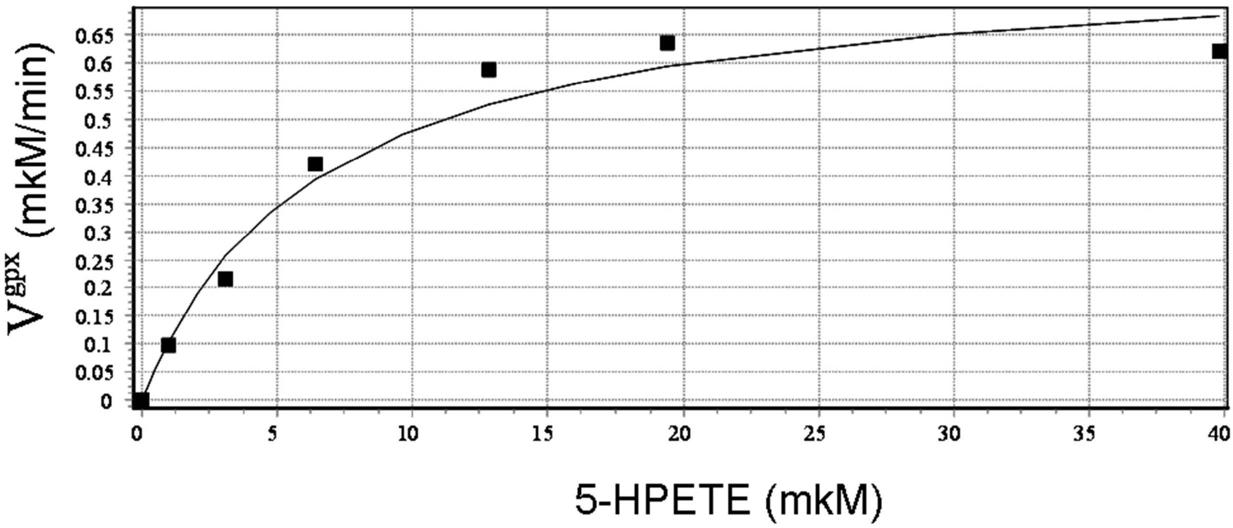

Figure A10.

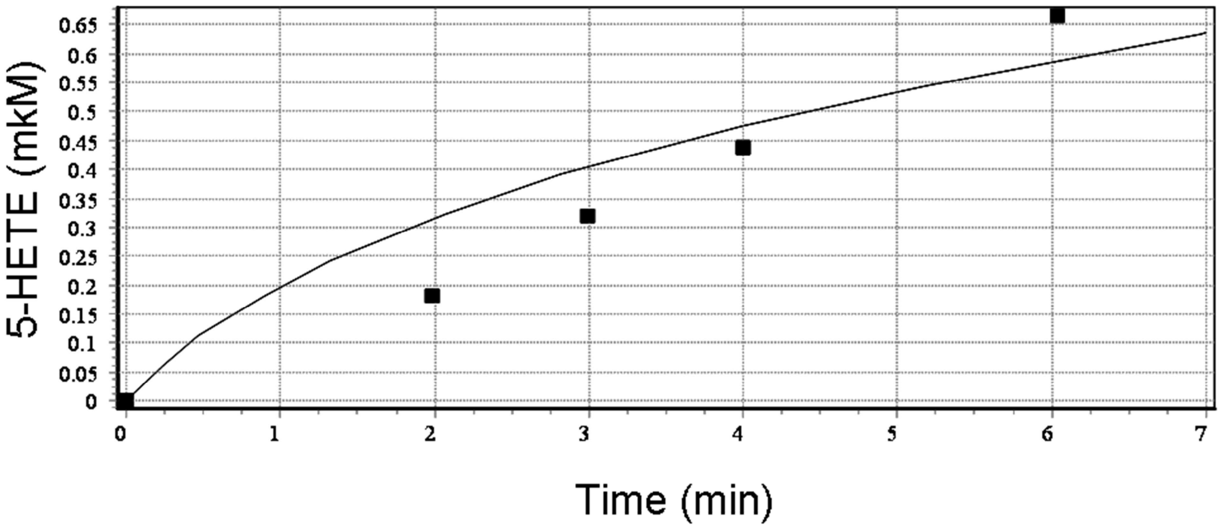

Figure A11

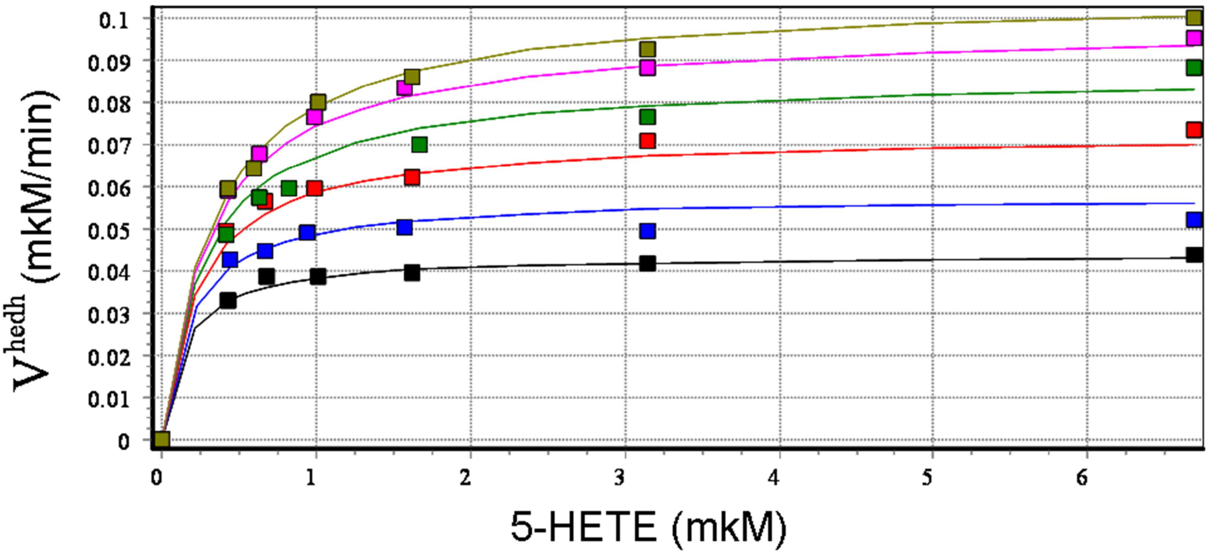

Figure A12

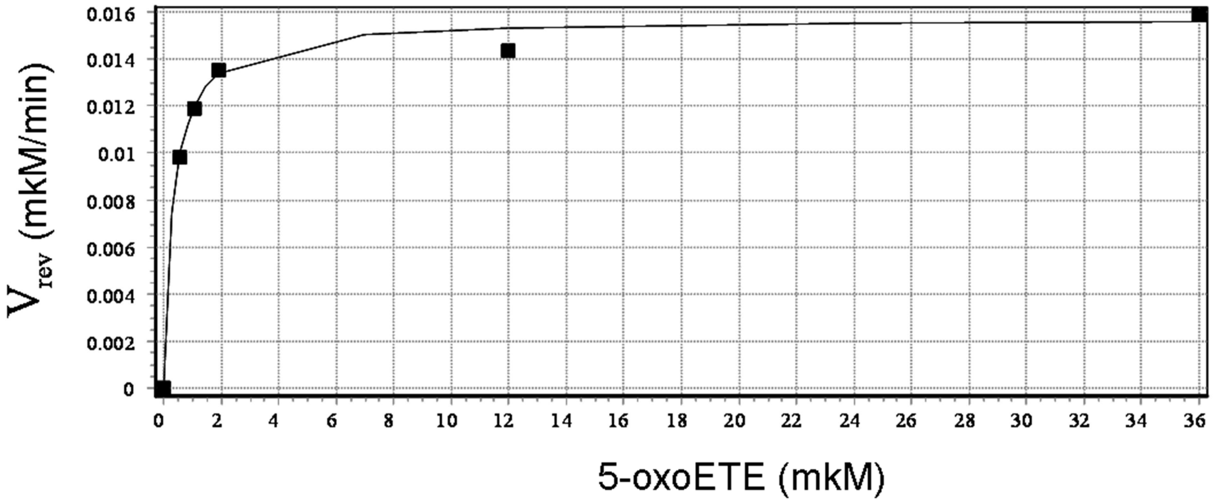
0.016
0.014
0.012
0.01
0.008
0.006
0.004
0.002
0
V_{rev} (mkM/min)
0
2
4
6
8
10
12
14
16
18
20
22
24
26
28
30
32
34
36
5-oxoETE (mkM)
